# The HexMaze: A Previous Knowledge Task on Map Learning for Mice

**DOI:** 10.1523/ENEURO.0554-20.2021

**Published:** 2021-08-10

**Authors:** Alejandra Alonso, Levan Bokeria, Jacqueline van der Meij, Anumita Samanta, Ronny Eichler, Ali Lotfi, Patrick Spooner, Irene Navarro Lobato, Lisa Genzel

**Affiliations:** 1 Donders Institute for Brain, Behaviour and Cognition, Radboud University, 6500 GL, Nijmegen, The Netherlands; 2 Centre of Cognitive and Neural Systems, University of Edinburgh, Edinburgh EH8 9JS, United Kingdom

**Keywords:** cognitive map, memory consolidation, navigation, schema, spatial

## Abstract

New information is rarely learned in isolation; instead, most of what we experience can be incorporated into or uses previous knowledge networks in some form. Previous knowledge in form of a cognitive map can facilitate knowledge acquisition and will influence how we learn new spatial information. Here, we developed a new spatial navigation task where food locations are learned in a large, gangway maze to test how mice learn a large spatial map over a longer time period—the HexMaze. Analyzing performance across sessions as well as on specific trials, we can show simple memory effects as well as multiple effects of previous knowledge of the map accelerating both online learning and performance increases over offline periods when incorporating new information. We could identify the following three main phases: (1) learning the initial goal location; (2) faster learning after 2 weeks when learning a new goal location; and then (3) the ability to express one-session learning, leading to long-term memory effect after 12 weeks. Importantly, we are the first to show that buildup of a spatial map is dependent on how much time passes, not how often the animal is trained.

## Significance Statement

While most tasks in human behavioral research are based on and embedded in familiar efforts and environments, rodents tend to be naive to the behavioral tasks and can draw only little benefit from previous experience. We developed a new task that can investigate the effect of previous knowledge on new memory acquisition. Within the task, we can differentiate between different previous knowledge effects. We show that different phases in this task are suitable for different approaches to memory: from simple reference memory to rapid consolidation once a map is established. Further, we show that building up a knowledge network is dependent on how much time passes and not how much training an animal receives.

## Introduction

How does one learn new spatial environment? And once a spatial layout of an environment is learned, how is it used when incorporating new information? After infancy, we rarely acquire new information in isolation; instead, most of what we learn throughout our lives can be associated with previous knowledge. For example, Harlow (1949) described learning sets as the “learning to efficiently learn” process of generalizing previous experience in a class of problems to new problems of the same class. Further, schemas, as proposed by Bartlett (1932) and expanded on by Ghosh and Gilboa (2014), are associated network structures based on previous experience that expedite long-term memory. Previous knowledge can also affect spatial and map learning: the more experience you have with an environment, the easier it will be to navigate through it and learn new goal locations (GLs) within it. In the past decade, more research into how previous knowledge affects learning in rodents has been provided, but how mice learn a very large, complex environment over a longer time period has not been investigated so far. The present project aims to tackle the question of which steps map–knowledge affects learning and provides a large, comprehensive dataset on mice spatial navigation for others to use with 16 mice over ∼10 months with a total of +30 000 trials.

Outside laboratory settings, rodents will learn the spatial layout of their home environment with likely food and water resources as well as danger zones. Further, they will also learn the complex layout of their home burrow system. Surprisingly, laboratory tasks rarely tap into this spatial ability of learning large spatial environments. Further, most experiments using more complex spatial abilities have been done in rats and not mice. Rat burrows have been used to test for path integration and general navigation patterns (Zanforlin and Poli, 1970; Zuri and Terkel, 1996; Alyan and McNaughton, 1999), and mazes composed of four or more connected square environments have been used to test whether rats take novel shortcuts (Roberts et al., 2007; Grieves and Dudchenko, 2013). Less has been done with mice. The most prominent spatial task with mice is the star maze by Rondi-Reig et al. (2006) and Fouquet et al. (2013). The star maze is a circular gangway maze that has five arms going off the main circular path. However, the maze is generally used to test how animals learn one single goal location with either an allocentric strategy based on cue-related navigation or a motor sequence strategy based on body turns. This goal location remains stable during training, and no changes are introduced to the environment. How previous knowledge of the environment is used to incorporate new information, such as a goal location switch, has not been investigated so far.

The distinction between early spatial learning and the incorporation of new information once the original spatial map has been established is critical. How much previous knowledge exists when learning something new will influence the rate of learning and consolidation as well as neural underpinnings. Relevant brain areas can show a shift in the presence of previous knowledge (Wang and Morris, 2010; van Kesteren et al., 2012; Squire et al., 2015; Genzel and Wixted, 2017; Alonso et al., 2020). In human research, the previous knowledge effect has been long established (Bartlett, 1932), but it was not introduced to rodent research until the seminal study of the paired-associates task introducing the schema effect on system consolidation in rats (Tse et al., 2007). During the task, rats initially learn a small map of six flavor–location associations: they receive a flavored pellet in the start box and learn that more of the same flavored pellets can be found in one specific sand well in an open field environment. After learning six flavor–location pairs over 9 weeks creating a mental map of paired-associate locations or “schema,” this map can be updated with new flavor–location pairs. In a sequence of articles, it was shown that previous knowledge accelerates learning to a one-trial event as well as the rate of systems consolidation (i.e., the process of memories that are initially hippocampal dependent becoming hippocampal independent) from weeks to days (Tse et al., 2007; Bethus et al., 2010). Further, in addition to the hippocampus, the medial prefrontal cortex needs to be active during encoding for memories to last (Tse et al., 2011; Wang et al., 2012). In these experiments, the schema is based on the map of flavor locations and not simply on the rule that flavors will be associated with locations, as they could show in a critical control experiment with an unstable map. The involvement of the medial prefrontal cortex as a structure for the schema effect—the expedition of long-term memory—was then later confirmed in humans (van Kesteren et al., 2010a,b; Ghosh and Gilboa, 2014; van Buuren et al., 2014). How experience of a complex spatial map will influence navigation and new learning has been investigated in humans (Patai et al., 2019), but so far not in rodents. This is surprising, since the concept of a cognitive map representation in the brain is of long standing (O'Keefe and Nadel, 1978). With place cells in the hippocampus and grid cells in the entorhinal cortex, we have learned about the basic building blocks of how the cognitive map is coded in the brain (McNaughton et al., 2006; Moser et al., 2008). These same fundamental building blocks have been shown to also be harnessed for nonspatial memory representation and associations between these (Behrens et al., 2018). Therefore, map learning can be the ideal model for us to understand how we build up as well as update (UP) our knowledge systems.

In the present study, we aimed at developing a new behavioral task in which we focus on map learning of a larger environment and how mice can use this type of previous knowledge to navigate to and flexibly update information about goals. Further, in this task we can investigate the role of previous knowledge on new memory acquisition and consolidation across different time-points in training. To achieve this, it is important that during both initial buildup of the knowledge network as well as later updates, the difficulty of the task and thereby the cognitive load remain the same. Thus, we chose to train mice in a large environment to navigate to a single goal location. We expect to see different types of previous knowledge effects on the performance of the mice, reflected in the length of their navigational paths: learning the general task principles (static food location and allocentric navigation from different starting positions), enhancing memory encoding (increased performance on the second up to the last trial of a session), and enhancing memory consolidation (increased long-term memory and performance on the first trial of each session). To test how quickly new information can be incorporated into this map, we changed the goal locations every few sessions.

We could show that mice learn this complex spatial map in the following three main phases: (1) Learning the initial goal location; (2) faster learning after 2 weeks when learning a new goal location; and then finally (3) a third phase after 12 weeks to express one-session learning, leading to long-term memory. Importantly, the map buildup is dependent on how much time passes (weeks), not how often the animal is trained (training days). In addition to the enhancement of long-term memory after map acquisition, we can distinguish a simple memory effect, reflected by better performance across the first couple sessions of the first goal location. Furthermore, an initial learning set effect after 2 weeks of training is seen in the first goal location switch as well as a late learning set effect after 12 weeks of training. This initial learning set effect is not expressed in the first trial of a session (long-term memory and thus different from the previous described effect) but does facilitate the increase of overall session performance. Finally, focusing on later learning after 12 weeks, we could show that the degree of overlap with previous knowledge influences navigational performance on the first session of a change (i.e., how quickly new information could be incorporated online). Thus, the HexMaze task allows the distinguishing of four effects of previous knowledge on memory expressed across three phases in time, ranging from learning set to rapid consolidation and within-session updating. With this task, we can provide a very rich dataset (+30,000 individual trials) that allows the investigation of spatial navigation patterns of mice and how they develop within a session as well as across weeks of experience with the spatial map of the maze.

## Materials and Methods

### Subjects

Five cohorts with four male C57BL/6J mice in each (Charles River Laboratories), which were 2 months of age at arrival, were group housed in the Translational Neuroscience Unit of the Centraal Dierenlaboratorium at Radboud University (Nijmegen, Netherlands). They were kept on a 12 h light/dark cycle, and before training were food deprived overnight during the behavioral testing period. Weight was targeted to be at 90 to 85% of the estimated free-feeding weight of the animals. All animal protocols were approved by the Centrale Commissie Dierproeven (protocol #2016–014-018). The first cohort (coh 1) was used to establish general maze and task parameters, and was not included in the current analysis.

### HexMaze

**Figure 1.**
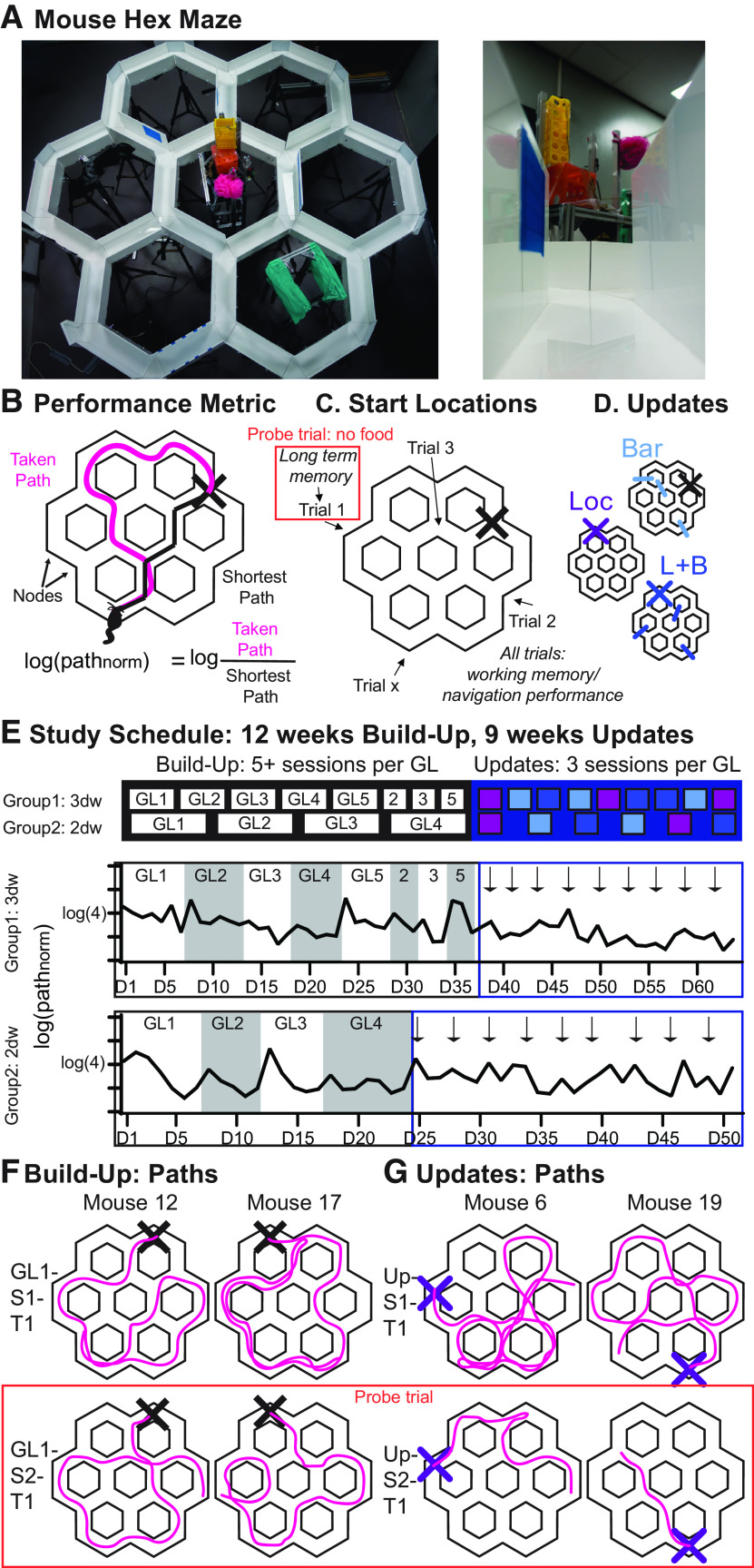
The Hex Maze. ***A***, Shows the maze with intramaze and extramaze cues (left) and the maze from the view of the mouse (right; also see Movie 1). ***B***, The main performance metric is the log-normalized path (path_norm_), with the lengths of the paths taken by the animal divided by the shortest possible path to the GL (indicated by the X). Thus, for all subsequent figures the number in brackets of the log is the relative length of the path taken by the animal, with 2 indicating that the path was twice as long as the shortest possible path. ***C***, During training, animals started each trial from a different location and had to navigate to a fixed GL. A first trial measures long-term memory performance and was used as a probe trial on critical sessions (no food present). Performance on all trials of the session measure general working memory/navigational performance in the known environment. ***D***, After the animals had acquired the general maze knowledge during the Build-Up, Updates were performed with inclusion of new Bars, new goal Locs, or the inclusion of L + B. ***E***, The general training schedule for all animals during the whole experiment. Animals were trained to one GL in a given session. For group 1, the GL was kept constant for seven sessions of GL1, then five or six sessions for GL2, and five or seven sessions each for GL3–5. Additionally, three of the initial five locations were repeated with each of three sessions. For group 2, the GL was kept constant for seven sessions of GL1, then five sessions for GL2–3, and seven sessions each for GL4. Finally, for all cohorts, each Update contained three sessions. The sequence of the Update types was counterbalanced across animals (session 1 of each update indicated with an arrow). Each Update type was repeated two to three times. Throughout all phases, the first trial of the second session and during Build-Up first trial of the fourth, fifth, or sixth session were used as probe trials. Group 1 was trained 3 d/week (3dw), group 2 was trained 2 d/week (2dw). ***F***, ***G***, Example paths of the Build-Up (***F***) and Updates (***G***) are shown (Movies 2, 3, 4, 5, 6, 7, 8, 9). T1, Trial 1. Data are in Extended Data Figure 1-1.

The HexMaze was assembled from 30 10-cm-wide opaque white acrylic gangways connected by 24 equilateral triangular intersection segments, resulting in a distance of 36.3 cm center-to-center between intersections (Fig. 1*A*). Gangways were enclosed by either 7.5- or 15-cm-tall white acrylic walls. Both local and global cues were applied to provide visual landmarks for navigation. Barriers consisted of transparent acrylic inserts tightly closing the space between walls and maze floor as well as clamped plates to prevent subjects bypassing barriers by climbing over the walls. The maze was held 70 cm above the floor to allow easy access by the experimenters.

10.1523/ENEURO.0554-20.2021.f1-1 Figure 1-1 Data from all trials used in all figures. Download Figure 1-1, XLSX file.

### Video acquisition and tracking

Two USB cameras (model C270, Logitech) were installed 2.1 m above the gangway plane with an overlapping field of vision (FOV) to provide full coverage of the arena and reduce obstruction of vision by maze walls. Image data (15 frames/s, 800 × 600 square pixels per camera) was acquired on a low-end consumer PC (Ubuntu version 19.04, AMD Ryzen 2200G processor, 8 GB RAM) with custom Python scripts (Anaconda Python version 3.7, OpenCV version 4.1.0) at controlled brightness and exposure levels. Images were immediately compressed and written to disk for offline analysis. In parallel, online tracking was applied for feedback to the experimenter and adjustments of the paradigm. Briefly, for each camera view a mask was generated at the beginning of the experiment based on the contrasting brightness of the maze and experimental room floor. This arena outline mask was applied to new frames, and a foreground mask was generated using the OpenCV MOG2 background estimation implementation (Zivkovic and van der Heijden, 2006). The resulting foreground mask was cleaned, and the centroid for the largest detected foreground object in a tracking search window was calculated as the putative location of the mouse in the maze. The location was smoothed over time using a Kalman filter, interpolating occasional occlusions by the maze walls and similar detection failure modes. The detected location was mapped to the closest node, and visually presented to the experimenter as well as logged for offline path analysis. Synchronization between cameras for offline analysis was enabled by presenting a blinking LED (1 Hz, 50% duty cycle) in the overlapping FOV of both cameras. Experimenters could indicate start and offset of trials using a remote presenter (model R400, Logitech).

### Behavioral training

After arrival and before training initiation, mice were handled in the housing room daily for 1 week (until animals freely climbed on the experimenter, see videos on https://www.genzellab.com/#/animal-handling/) and then habituated to the maze in two 1 h sessions (all four cage mates together) with intermittent handling for maze pickups (tubing; Gouveia and Hurst, 2017). Mice were trained either on Mondays, Wednesdays, and Fridays (coh 1–3, group 1) or Tuesday and Thursday (coh 4 and 5, group 2). Per training day (session), each mouse underwent 30 min of training in the maze, resulting in up to 30 trials per session. The maze was cleaned with 70% ethanol between animals (later clean wipes without alcohol to avoid damaging the acrylic and to encourage returning in the next trial), and a heap of food crumbles (Coco Pops, Kellogg’s) was placed at a previously determined GL, which varied for each animal. GLs were counterbalanced across animals, as well as within animals across GL switches (e.g., one of four animals), and one of four GLs per animal would be located on the inner ring of the maze while the others were on the outer ring (to shape animal behavior against circling behavior). Start locations for each day were generated based on their relation to the GL and previous start locations (locations did not repeat in subsequent trials, and at least 60% of the trials had only one shortest path possible, the first trial was different from the last and first trial of the previous session, and locations had at least two choice point distances to each other as well as the GL). On average, 30 start locations, which were generated the day before training, were needed per day per mouse. After the mouse reached the food and ate a reward, the animal would be manually picked up with a tube, carried around the maze to disorient the mouse, and placed at the new start location. All pickups in the maze were performed by tubing (Gouveia and Hurst, 2017). After placing the animal at the start location, the experimenter quickly but calmly moved behind a black curtain next to the maze to not be visible to the animal during training trials. Each cohort had multiple experimenters (bachelor and master interns, both female and male experimenters), and different cohorts were run by different sets of students. Each mouse was habituated to each experimenter before training in the maze. Each training day, the animals were brought to the training room at least 20 min before training start.

Training consisted of two blocks: Build-Up and Updates. During probe sessions [each second session of a GL switch and additionally in Build-Up; GL1, session 6 (S6); GL2, S5; GL3–5, S4], there was no food in the maze for the first trial of the day and each time for the first 60 s of the trial to ensure that olfactory cues did not facilitate navigation to the GL. After 60 s, food was placed in the GL while the animal was in a different part of the maze (to avoid the animal seeing the placement). All other trials of the day were run with food at the GL. Probe trial and GL switches were initially minimized to help shape the animal behavior. In the first trial of the day, animals would not find food at the last presented location for both the first session of a new GL as well as probe trial days (e.g., always the second session of a new GL); thus, these sessions were interleaved with normal training sessions with food present at the last known location in the first trial of the day to avoid the animals learning the rule that food is initially not provided.

To measure the performance of the animals, the actual path a mouse took was divided by the shortest possible path between a given start location and the GL, resulting in the log of normalized path length (Fig. 1*B*) and functioning as a score value. Given a sufficient food motivation and an established knowledge network of the maze, a mouse should navigate the maze efficiently. A score of 0 indicated that the mouse chose the shortest path and navigated directly to the goal. On average, animals would improve from a 3 times to a 1.5–2 times longer path length than the shortest path, corresponding to 0.4 and 0.2–3 log values. Random walks (random choice at each node) through the maze are estimated with a model to result in a 4 times longer path (0.6 in log). A more refined random walk with random choices at each node and once in a while a long diagonal run are included in the companion article (Vallianatou et al., 2020). The normalized path length of any first trial of a session was used to measure long-term memory since training sessions were 2–3 d apart.

The first trials of the second sessions (probe trials) of each goal location in Build-Up and Update phase were watched to score the number of times that animals crossed their current and previous goal location as well as the amount of time they dwelled there. As a control, the same method was applied to two other nodes, one on the inner ring of the maze and the other on the outer ring. These nodes were selected in such a way that they were not close to each other and to the goal locations, with at least three gangways between them. Further, to control a false-positive result, nodes that were in the way between goal locations were not chosen as a control.

Food motivation was ensured by restricting access to food for 8–24 h before training and confirmed by both the number of trials run each day as well as the count of trials during which the animal ate food at the first encounter with the food in each trial. If animals were not sufficiently motivated, the count of both would decrease. Additionally, animals were weighted three times a week and the average weekly weight was ensured to not fall below an estimated 85% free-feeding weight, which was adapted for the normal growth of each animal across time.

### Data analysis

The normalized path length for all trials was calculated using MATLAB 2017b (MathWorks). Repeated-measures ANOVAs were run in SPSS Statistics 25 (IBM) to determine the effect of goal location switches and session on the log-normalized path length during the Build-Up and across the three different types of Updates. Within-subject factors were goal location, update type, session and trial. The only between-subject factor was training 2 d/week (group 2) versus training 3 d/week (group 1). If sphericity was not given, the Greenhouse–Geisser correction was used.

## Results

### The HexMaze

The HexMaze is arranged as six regular densely packed hexagons, forming 12 two-way and 12 three-way choice points (nodes) 36.3 cm apart, in total spanning 2 × 1.9 m (Fig. 1*A*). Gangways between nodes were 10 cm wide and flanked by either 7.5- or 15-cm-tall walls. Maze floor and walls were white and opaque, with local and global cues applied in and around the maze to enable easy spatial differentiation and good spatial orientation, leading, overall, to a complex, integrated maze. During training, food was placed in one of the nodes and the animal had to learn to navigate efficiently from different start locations to the goal location. To measure performance in this maze, we divided the taken path of each trial by the shortest possible path (Fig. 1*B*; for comparison of different performance parameters see below; see also Fig. 9). To eliminate the resulting skewness (skewness, 3.33), we used the log of the normalized path (skewness, 0.72). The reason for the skew of the data is that ∼30% of the trials are direct runs (paths), resulting in values of 1 and 0 (without and with log, respectively), and animals cannot perform better than a direct run (i.e., there is a ceiling effect and maximum values for best memory performance). Thus, no normal distribution can be achieved with this type of data. Using the log decreases the skew and allows for use of GLM in analysis. However, the data without log (see last section in Results; see also Fig. 9) show the same learning curves and effects. Each session lasted 30 min per animal, resulting in 25–35 trials per session with each trial starting from a different location within the maze (Fig. 1*C*). Evaluation of the performances of only the first trials of the sessions measures long-term memory performance, and during critical sessions (e.g., the second session of a new GL), to measure long-term memory after one session learning, this first trial was used as a probe trial where the food reward was not present for the initial 60 s to control for olfactory cues. In contrast to the first-trial evaluation for long-term memory, looking at the performance over all trials gives a measure of the overall working memory and navigational performance within the environment.

Animals went through the following two phases of training: Build-Up and Updates. In the Build-Up, the animals should create a cognitive map of the maze environment; in contrast, during Updates, stable performance is achieved, and they should be simply updating the cognitive map. These two phases also differed in the frequency of GL switches: during Build-Up, the GL remained stable for five and more sessions, while during Updates a change occurred every three sessions (see also below). Different Update types were performed, including barriers in the environment (Bar), changing the goal location (Loc), and doing both (L + B; Fig. 1*D*).

Five cohorts (coh 1–5) of four animals each were trained in the maze (Fig. 1*E*). Coh 1 was a pilot cohort to establish maze size, food deprivation, and other parameters, and is not included in the data. Group 1 (coh 2 and 3) was trained three times a week, while group 2 (coh 4 and 5) was trained two times a week. The GL was switched during the Build-Up every five to seven sessions (GL1, seven sessions; GL2, five of six sessions; GL3–5, five of seven sessions) to test when rapid updating could occur. Faster switches were initially avoided, to help shape the behavior of the animal. In the first trial of the day, animals would not find food at the last presented location for both the first session of a new GL as well as probe trial days (e.g., always the second session of a new GL); thus, these sessions were interleaved with normal training sessions with food present in the first trial at the last known location to avoid the animals learning the rule that food is initially not provided.

After 12 weeks of Build-Up, all groups were tested in the Updates, where a change (given by the different Update types) was introduced every three sessions. The sequence of the different Update types (Loc, Bar, L + B) was counterbalanced across repetition and cohorts. Further, the GLs were also counterbalanced across animals within a cohort as well as across cohorts. To ensure that the identity of individual GLs did not account for learning effects over time, the sequence was reversed between cohorts (e.g., GL1 of the first animal in coh 2 would be GL5 of the first animal in coh 3).

Overall performance for each group across time can be seen in Figure 1*E*. Different learning effects were found as highlighted in individual paths (Fig. 1*F*,*G*, Movies 1, 2, 3, 4, 5, 6, 7, 8, 9), as follows: on the first trial of the first training day of the Build-Up, the animals show random movement through the maze and just by chance find the GL (Fig. 1*F*, Movies 2, 4). On the next day at the first trial, some but not all animals already show more goal-directed behavior (Fig. 1*F*, Movies 3, 5). In contrast, during the Updates on the first trial of a new GL, the animals still show random exploration, since the goal location is unknown, but are then more likely to show memory effects and goal-oriented behavior in earlier trials of session 1 (Fig. 1*G*, Movies 6, 8). And in the succeeding session of the Updates most animals had already shown more goal-oriented navigation to the reward location on the first trial (Fig. 1*G*, Movies 7, 9).

Movie 1. Mimicking animal view in the maze. 10.1523/ENEURO.0554-20.2021.video.1

Movie 2. Three trials of S1 during the Build-Up Mouse 12. 10.1523/ENEURO.0554-20.2021.video.2

Movie 3. Three trials of S2 during the Build-Up Mouse 12. 10.1523/ENEURO.0554-20.2021.video.3

Movie 4. Three trials of S1 during the Build-Up Mouse 17. 10.1523/ENEURO.0554-20.2021.video.4

Movie 5. Three trials of S2 during the Build-Up Mouse 17. 10.1523/ENEURO.0554-20.2021.video.5

Movie 6. Three trials of S1 during the Update Mouse 6. 10.1523/ENEURO.0554-20.2021.video.6

Movie 7. Three trials of S2 during the Update Mouse 6. 10.1523/ENEURO.0554-20.2021.video.7

Movie 8. Three trials of S1 during the Update Mouse 19. 10.1523/ENEURO.0554-20.2021.video.8

Movie 9. Three trials of S2 during the Update Mouse 19. 10.1523/ENEURO.0554-20.2021.video.9

### Building and updating the map

**Figure 2.**
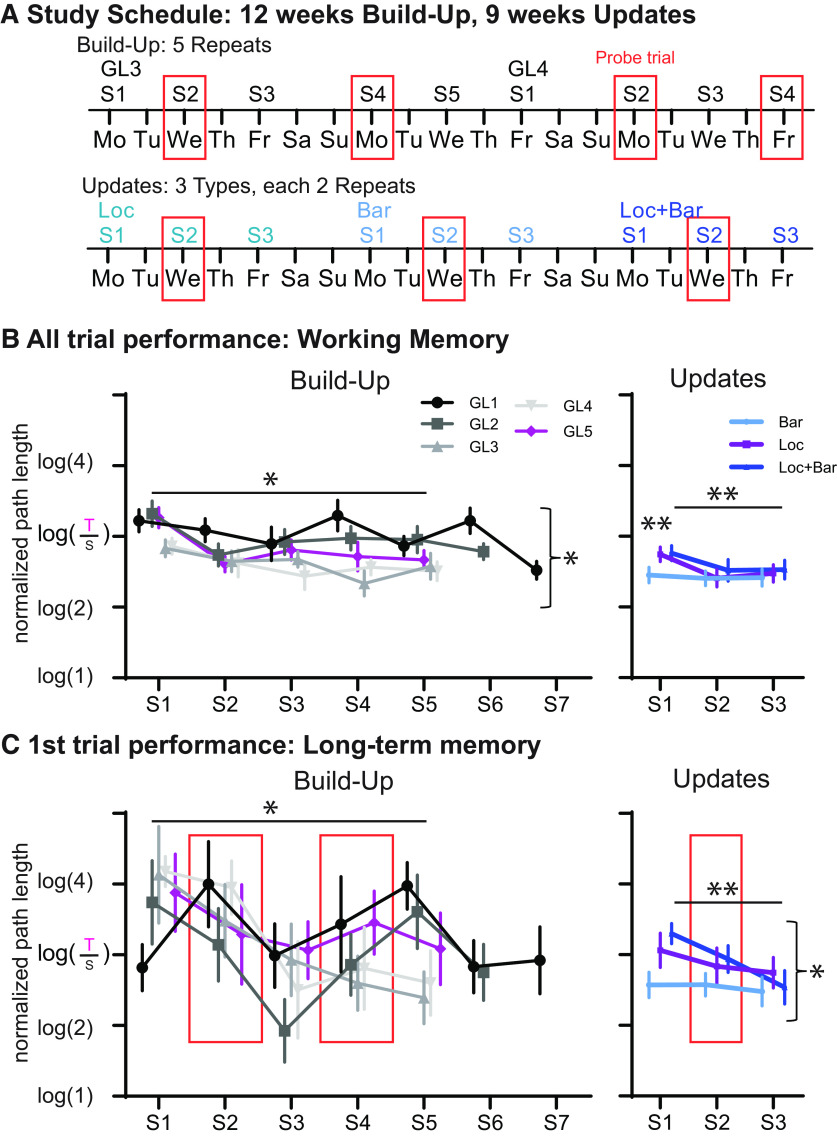
HexMaze performance group 1 3 d/week training. ***A***, Shows schedule examples for the Build-Ups and Updates. Orange boxes indicate days with probe trials (no food for 60 s of the first trial). ***B***, Performance across all trials (including first trial) measures general working memory/navigational performance within the environment. During Build-Up, there was a significant effect across session and across the five GL switches. In contrast, during Updates, only if a location switch was involved in the update (Loc/L + B), performance was worse during the first session of the change and an improvement across sessions is visible. ***C***, Performance on the first trial of each session measures the ability to remember the GL from 2–3 d ago. During the Build-Up, long-term memory improved across sessions. During the Updates, there was an improvement across sessions as well as a difference between types with larger changes in the environment (linear from Bar to both L + B), leading to worse performance. This is especially noticeable in session 1 for Loc and L + B switches where the goal is initially unknown, whereas for a Bar update only an adaptation of the route is involved. *p < 0.05, **p < 0.01. Error bars are the SEM. The number in brackets of the log is the relative length of the path taken by the animal (taken path T/shortest path S), with 2 indicating that the path was twice as long as the shortest possible path.

To formally investigate the effects seen in the individual paths, we analyzed group-level performance in more detail. Group 1 (total, *n* = 8) was trained Monday/Wednesday/Friday (Fig. 2*A*, example study schedule) and during the Build-Up showed a significant improvement in navigation to the GL (all trials, which includes the first trial) across sessions as well as across GLs (GL1–5; session: *F*_(4,28)_ = 6.2, *p* = 0.001; GL: *F*_(4,28)_ = 3.3, *p* = 0.026; interaction, *F*_(16,112)_ = 1.4, *p* = 0.15). For both session and GL, the linear contrast was significant (session, *p* = 0.027; GL, *p* = 0.043; Fig. 2*B*). In the Updates, the animals overall performed better than in the Build-Up (*F*_(1,7)_ = 8.2, *p* = 0.024) and continued to show a significant improvement of performance over the three sessions (session: *F*_(2,14)_ = 12.9, *p* = 0.001; linear contrast, *p* = 0.005). Additionally, there was an effect of Update type (Bar, Loc, L + B) as well as a type × session interaction: in contrast to the Update types with location changes, animals already performed well in session 1 of the barrier updates (type: *F*_(2,14)_ = 3.5, *p* = 0.058; with linear contrast across Bar, Loc, and L + B, *p* = 0.027; interaction: *F*_(4,28)_ = 2.6, *p* = 0.059; orthogonal comparison session 1 Bar vs Loc/L + B, *p* = 0.01). During the first trial of each session, the animal had to rely on long-term memory (2–3 d between sessions) to navigate to the current GL. To minimize olfactory cues (e.g., chocolate smell and markings), the maze was cleaned with alcohol between animals, further on critical sessions (e.g., second session after a change to test for one-session learning), and no food was present in the maze for 60 s during the first trial. These probe trials were performed in sessions 2 and 4/5 or 6 during the Build-Up and in session 2 during the Updates (Fig. 2*A*). Across sessions, long-term memory improved independent of the GL during the Build-Up (Fig. 2*C* ; session: *F*_(4,28)_ = 4.0, *p* = 0.01; linear contrast, *p* = 0.056; GL: *F*_(4,28)_ = 0.4, *p* = 0.77; interaction: *F*_(16,112)_ = 1.1, *p* = 0.34). In the Updates, long-term memory increased across sessions as well as differed between Update types (session: *F*_(2,14)_ = 3.7, *p* = 0.053; with linear contrast, *p* = 0.009; type: *F*_(2,14)_ = 3.7, *p* = 0.052; with linear contrast across Bar, Loc, L + B, *p* = 0.028; interaction: *F*_(4,28)_ = 0.58, *p* = 0.68). Similar to the all-trials performance, in barrier Updates performance was better than in the other two types of Updates where the GL changed.

### Time versus training

**Figure 3.**
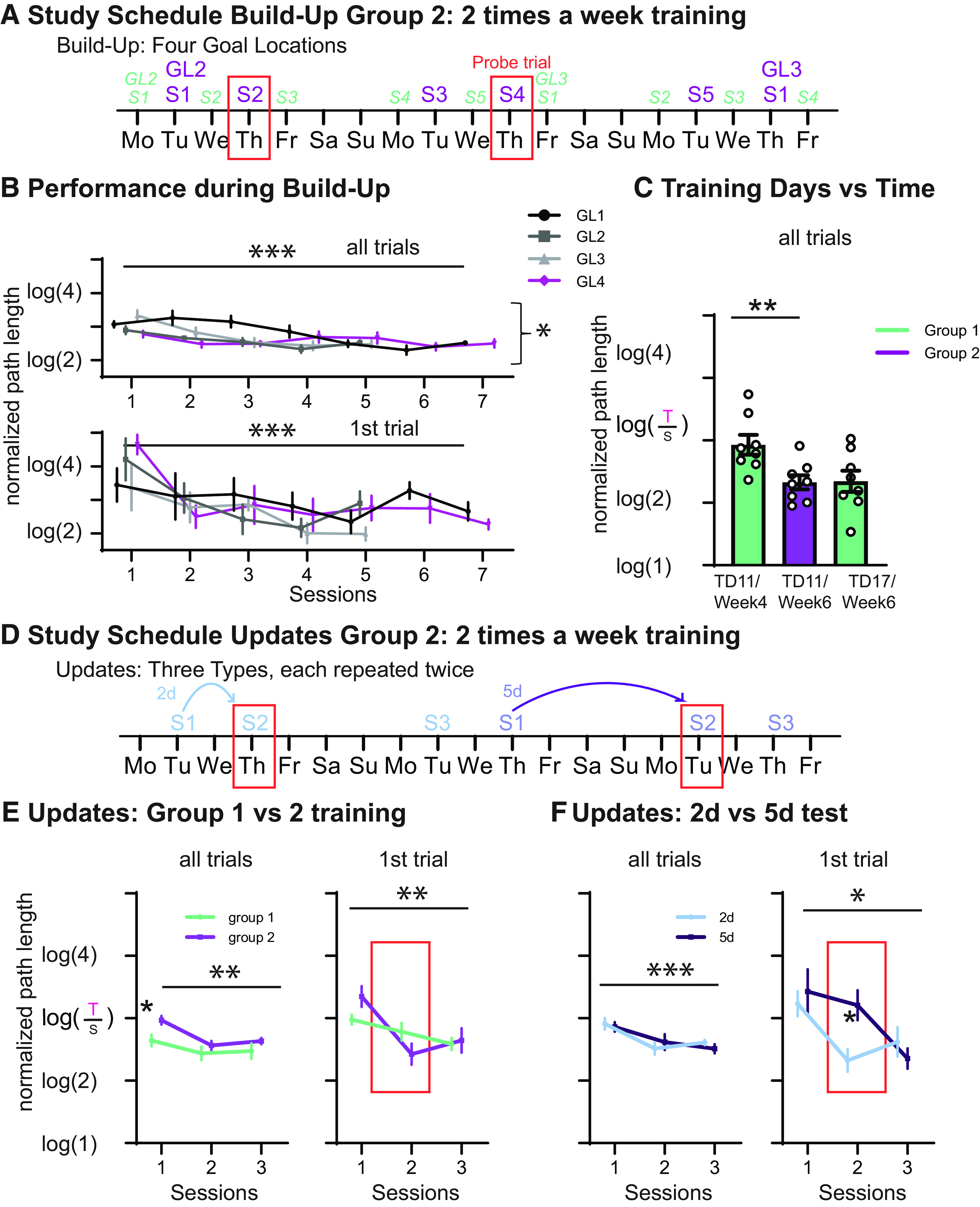
HexMaze group 2 performance 2 d/week training. ***A***, Shows schedule examples for the Build-Up. The schedule for group 2 is shown in purple, and for group 1 in light green, illustrating the resulting shift in alignment of training days and time. Orange boxes indicate days with probe trials (food not present for 60 s of the first trial). ***B***, Performance across both the all-trials measurement (general working memory/navigational performance within the environment) and the first-trial measurement (long-term memory). We found a significant improvement in performance across sessions for both measures and additionally across GL and GL × session interaction for all trials. ***C***, To compare 2 with 3 d/week training, we included the corresponding training day as well as session according to the time of group 1 and compared these with the performance of group 2. It is important to note, that the performance depended on how much time had elapsed since first exposure to the maze (weeks), not how much training the animals had received (TD, training day). ***D***, Examples from the study schedule of the Updates. With 2 d/week a natural alternation of 2 and 5 d, gaps ensued during the Updates. ***E***, Comparing only the 2 d Updates of group 2 with the Updates of group 1 (also 2 d gaps) showed only an Update difference during the first session. ***F***, Plotted is the performance during Updates for group 2 for both the 2 and 5 d delays. One session of training only led to significant long-term memory that lasted 2 d not 5 d, whereas two training sessions did indeed lead to a 5 d memory persistence visible in the third session (2 d condition for session 2). *p < 0.05, **p < 0.01, ***p < 0.001. Error bars are the SEM. The number in brackets of the log is the relative length of the path taken [taken path (T)/shortest path (S)] by the animal, with 2 indicating that the path was twice as long as the shortest possible path.

In contrast to group 1, group 2 (*n* = 8) were trained only 2 d/week, which resulted in a shift between the training day and the time alignment between both groups (Fig. 3*A*). As with 3 d/week training, 2 d/week training lead to an improvement in all-trials measurement across sessions as well as across GLs (GL1–4; session: *F*_(4,28)_ = 18.3, *p* < 0.001; linear contrast, *p* < 0.001; GL: *F*_(3,21)_ = 4.7, *p* = 0.011; linear contrast, *p* = 0.044); further, in contrast to the 3 d/week training, there was a session × GL interaction (*F*_(12,84)_ = 2.7, *p* = 0.004) with a faster improvement across sessions in later GLs (Fig. 3*B*). Long-term memory (first-trial performance) improved across sessions (session: *F*_(4,28)_ = 12.5, *p* < 0.001; linear contrast, *p* = 0.001), but there was no change from one GL to the next (*p* = 0.49), as also seen with group 1. Including both group 2 and group 1 in one ANOVA revealed a GL × session × training type interaction for all trials (*F*_(12,168)_ = 1.9, *p* = 0.039), and for first trials it revealed a training-type main effect (*F*_(1,13)_ = 6.7, *p* = 0.023) as well as a marginal session × training type interaction (*F*_(4,52)_ = 2.4, *p* = 0.066).

As one of the goals was to evaluate whether general performance was determined by the amount of time that had passed in contrast to how much training the animals had received, we included the same training day of group 1 and group 2 as well as the session of group 1 that corresponded to the same week of training as group 2 in a univariate analysis (*F*_(2,21)_ = 5.253, *p* = 0.014; group 1: training day 11, session 4 of GL2, during week 4 and training day 17; session 4 of GL3, during week 6; group 2: training day 11, session 4 of GL2, during week 6). These specific sessions were chosen, since only then did the same session number (here, session 4) occur at the same time in weeks as well as the day within the week across groups; thus, it was the only training day that could compare time versus training overall but could still control for the amount of training to the current goal location as well as how long ago the last training session was performed. Group 2 performed in a similar manner to group 1 when compared with how much time had elapsed, but was significantly better than group 1 with the same amount of training (Fig. 3*C*). Thus, performance in the HexMaze was more dependent on the time period in which the animals had been exposed to the maze and not how much training or exposure itself was involved.

To further validate whether this also applies to the previous knowledge effects, we focused as a next step on the Updates (Fig. 3*D*). Both the all-trial as well as first-trial measure showed an improvement across sessions (*F*_(2,28)_ = 9.5, *p* = 0.001; with linear contrast, *p* = 0.005) as well as a marginal session × training interaction (*F*_(2,28)_ = 3.1, *p* = 0.06), but did not expose an effect of training amount (*p* = 0.87; Fig. 3*E*). Only during the first session did group 2 perform worse than group 1 (*p* = 0.01). Thus, despite the decreased amount of training, rapid updating was still possible, indicating that the creation of a cognitive map is dependent on time, not on training.

The 2 d/week training schedule also allowed us to investigate how many sessions are necessary for memory persistence as the training schedule naturally alternated with 2 and 5 d gaps between sessions (Fig. 3*D*). While one session was sufficient for the animals to remember where the food was located in the first trial 2 d later, this memory did not last 5 d (Fig. 3*F*). However, after two sessions of training (Fig. 3*F*, 2 d condition), the animals did remember the GL in the third session (5 d after the second session; session: *F*_(2,14)_ = 8.1, *p* = 0.005; with linear contrast, *p* = 0.016; interaction session × delay: *F*_(2,14)_ = 3.6, *p* = 0.054; delay overall, *p* = 0.34). In contrast, general navigational performance (an all-trial measure) did not show a difference between the two delays (interaction, *p* = 0.24; delay, *p* = 0.9; session: *F*_(2,14)_ = 34.7, *p* < 0.001; with linear contrast, *p* < 0.001).

### Three phases of map learning

**Figure 4.**
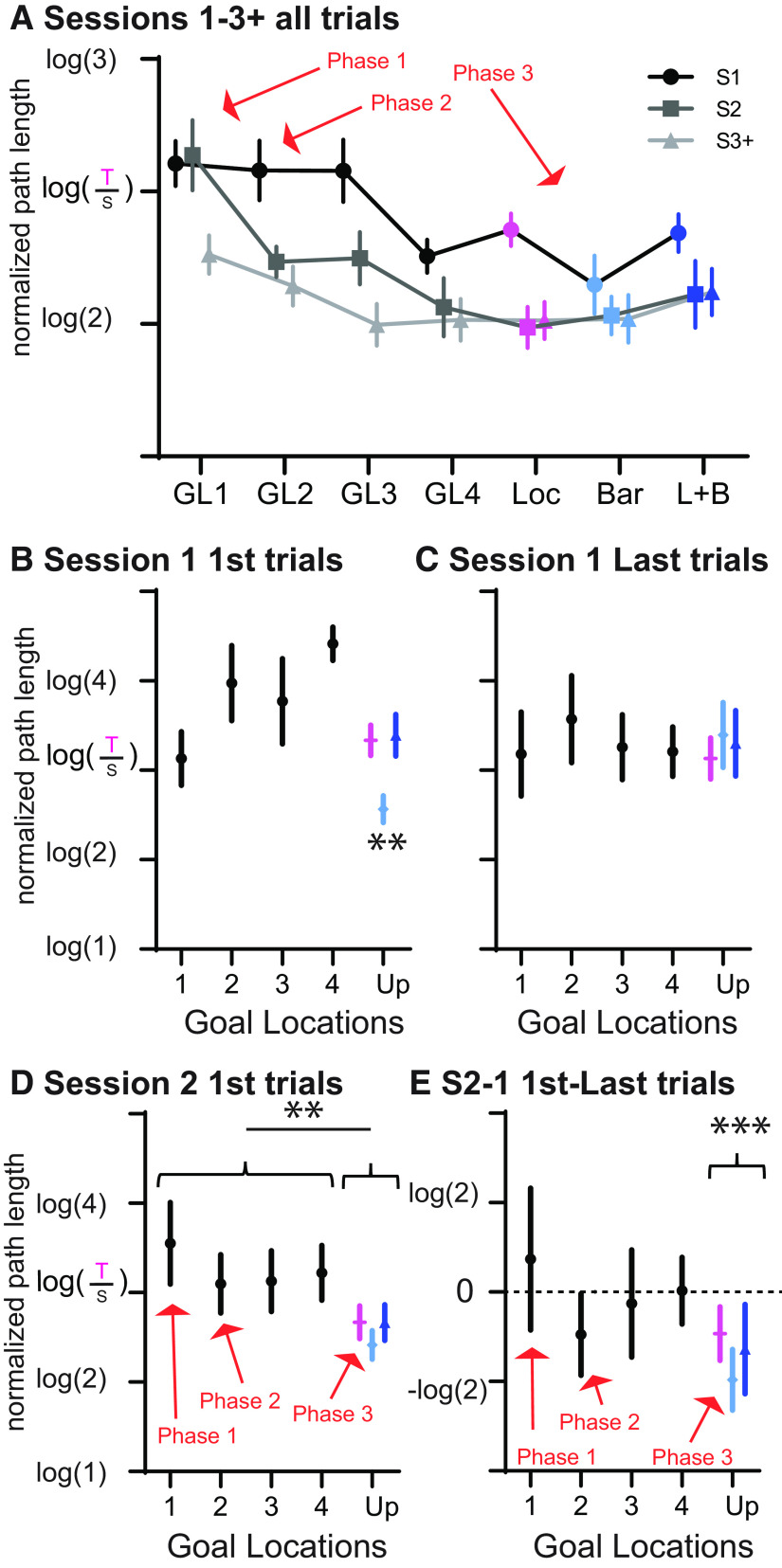
Three phases of map learning. ***A***, Plotted are all trials separated for the four GLs during Build-Up as well as the different Update types with separate lines for first session, second session and third session onwards (for Build-Up, it is S3–5/7; for Updates, it is just S3 since no further sessions were run). Three learning phases are noticeable: learning the first goal location, learning the second goal location with better session 2 performance, and learning the Updates with already good session 1 performance. ***B–E***, The first trial of session 1 (***B***), the last trials of session 1 (***C***), the first trials of session 2 (***D***), and the change from the last trial of sessions 1 to the first trials of session 2 (***E***) are shown for the different goal locations during Build-Up (GL1–4 as well as the different update types). The first trial performance during session 1, when the goal is unknown, first became worse in GL2–4 compared with GL1, most likely because of animals first navigating to the old goal location. Only in the Barrier updates (light blue) was performance better than in all other GLs and updates since the location did not change. At the end of session 1 (last trial), there is no difference between the different GLs and updates. The three phases of learning are again noticeable in the first trial of session 2, reflecting long-term memory after one session of training. This showed a stepwise function, improving in GL2–4 in contrast to GL1 and improving even more during the updates. The same is reflected in the difference values presented in ***E*** (updates; one-sample *t* test to 0: *t*_(71)_ = 4.2, *p* < 0.001).

Combining the data from groups 1 and 2, let us delve further into different phases of map learning. The main difference between the learning phases is how quickly the animals can adapt their performance to new information (e.g., a new goal location). First, all-trial performance was evaluated and separated for the four goal locations during Build-Up and the different Update types, and each of these for sessions 1, 2, and 3+ (sessions 3–5/7 for Build-Up, only session 3 for Updates since no other sessions were run). This analysis highlights three phases of learning (Fig. 4*A*; GL/UP: *F*_(6,90)_ = 4.7, *p* < 0.001; session: *F*_(2,30)_ = 40.1, *p* < 0.001; GL/UP × session interaction: *F*_(5.4,81.6)_ = 2.8, *p* = 0.018). When learning the first goal location, the animals need three and more sessions to reach good performance (phase 1). In contrast, when learning the second goal location, the animals already perform better at the second session (phase 2). Finally, during GL4 and the Updates, the animals already perform better in the first session but also have additional gains to the second session (phase 3). Importantly, already in the first few goal locations during Build-Up the animals reach their best possible performance in the later sessions. The difference to the Update phase is that during Build-Up it takes more sessions to reach that optimal performance level. Once the animals reach the Update phase, performance is stable. Therefore, the different phases in map learning are expressed in how quickly they can adapt to new goal locations and are not confounded by a general, continuous increase in performance.

To further focus on changes in the first two sessions across the different learning phases, the first and last trial of session 1 of a change and the first trial of session 2 are plotted for the different goal locations in the Build-Up and Updates (Fig. 4*B-E*). The first trial of session 1 was consistently high across all phases with the exception of the barrier updates, reflecting the fact that only in that Update type was the current goal location known and that the first trial did not represent a search for the new goal location (*F*_(1,112)_ = 6.5, *p* < 0.001; orthogonal comparison barrier vs other, *p* = 0.0018). The final trial of session 1 was also quite consistently stable across all phases, emphasizing that gains because of within-session learning also remained similar across phases (*F*_(1,112)_ = 0.1, *p* = 0.96). The main difference among the three learning phases can be seen in the first trial of the second session, reflecting long-term memory after one-session learning (*F*_(1,112)_ = 3.6, *p* = 0.017; orthogonal comparison Build-Up and Updates, *p* = 0.0037). There were gains from the very first goal location to subsequent goal locations during Build-Up but even more gains during the Updates, highlighting the stepwise increase in long-term memory performance over the different learning phases. These gains are also reflected when comparing performance on the last trial of session 1 to the first trial of session 2 (Fig. 4*E*). In the first goal location of Build-Up, this metric is positive, reflecting worse performance after the 24 h break, while during the other goal locations of Build-Up it is approximately zero, showing that they sustain their final performance level across the offline period. In the Updates, negative values are seen (one-sample *t* test to 0: *t*_(71)_ = 4.2, *p* < 0.001), which shows that they perform even better at the first trial of the second session compared with the final trial of the first session, thus showing an offline gain in performance.

In sum, there seem to be three phases in map learning: (1) learning the new goal location; (2) learning the second goal location 2 weeks later, when performance gains close to optimal performance are already seen in the second session but are not yet expressed in long-term memory (first trial of the second session); and finally, (3) after 12 weeks, when performance gains are already expressed in the first session to a new goal location and also translate to long-term memory effects with good performance at the first trial of the second session. This analysis also helps to distinguish among task–rule learning (e.g., “I need to run to a goal location”), maze learning (maze layout and surrounding cues), and goal learning (where in the maze is the food). In Figure 4*E*, the amount of training for one goal is controlled for, thus excluding the general effect of goal learning (for each data point, the amount of exposure to the current goal is the same: one session). The general task should be learned by the animal by GL2 or at the latest by GL3 (by then the animal learned that goal locations can change). Thus, only maze layout learning can explain the additional benefit seen in the Updates.

### Previous knowledge effects

**Figure 5.**
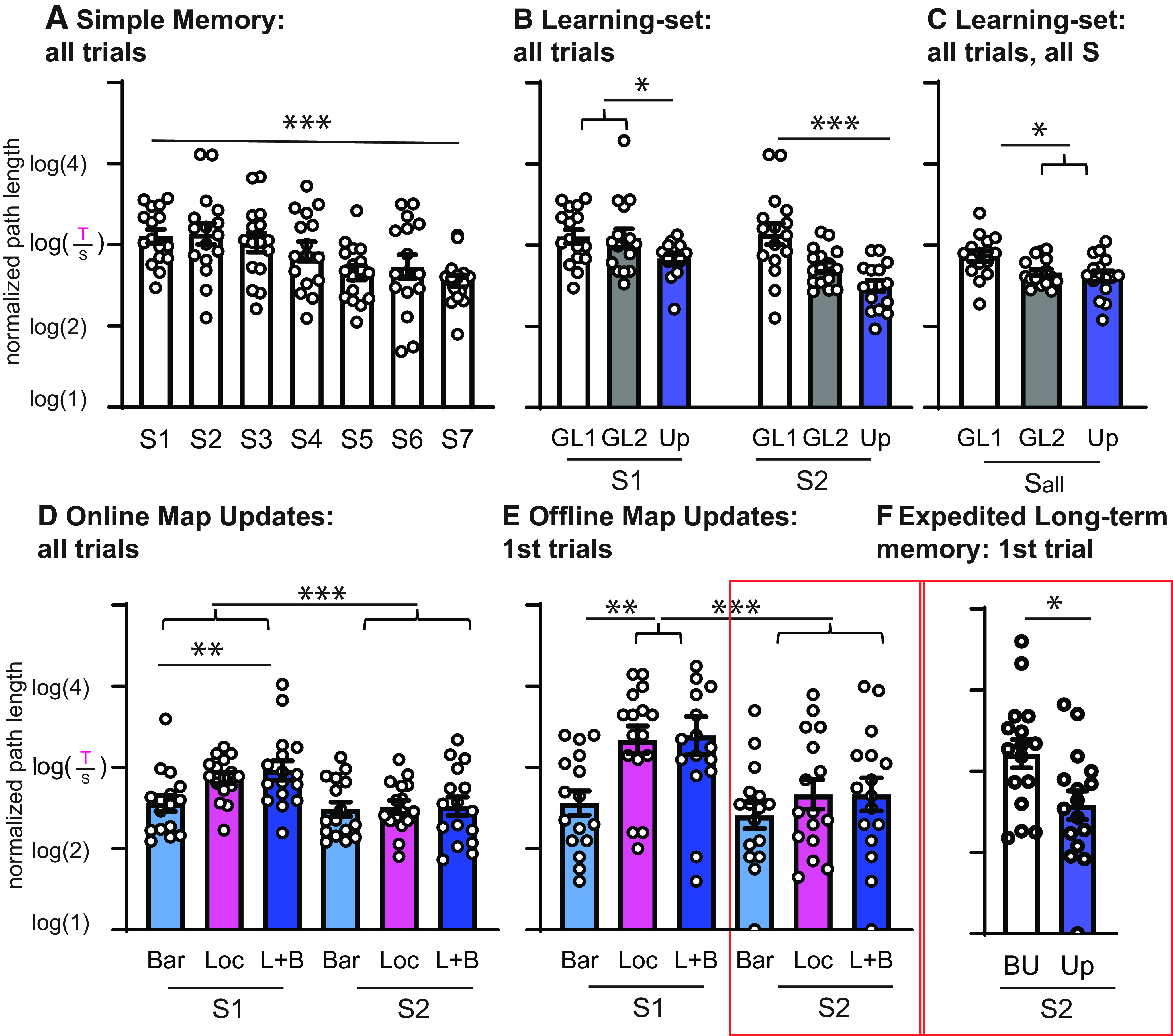
Previous knowledge effects. In these panels, we highlight some previous knowledge effects ***A***, The whole session performance for the first GL during the Build-Up. The significant session effect reveals a performance increase dependent on experience, indicating a more efficient working memory/navigational performance. ***B***, Plots the performance for the first two sessions of the first two GLs during the Build-Up, as well as Updates (averaged for all types). Already for the second GL (3 weeks since training start), a significant increase in performance (decrease of path length) is seen in the second session compared with the first session. This overnight (offline) performance increase is comparable to the increase found after seven sessions for the first GL. This may represent a more efficient consolidation and updating effect, but is expressed only in the whole session average (not long-term memory present in the first trial; Figs. 2, 3). During the Updates, this performance increase is already visible in the first session with additional offline gains found in the second session. This three-step performance gain is reminiscent of a learning-set effect (Harlow, 1949). ***C***, Considering all sessions, we find that animals already reach overall plateau performance by the second GL. ***D***, Zooming in on the performance during the first and second session during the Updates, another previous knowledge effect is revealed across the different Update types. The Bar, Loc, and L + B differed in their overlap of previous knowledge (or need for updating that knowledge), which influenced how well they performed (all-trial) in the first session. ***E***, The same effect but now only for the first trials. Only in the presence of a goal switch did performance in the first session decrease. However, by the second session this performance difference was gone, revealing that one session is sufficient for the memory update. ***F***, The performance of only the first trial of the second session during the Build-Up and Updates (only Loc and L + B) where long-term memory (2–3 d) after one session of learning a new GL improves from Build-Up to Updates. Thus, it seems that once a cognitive map is established, only one session of training leads to better long-term memory performance. Orange boxes indicate that the trial was used as a probe trial, meaning food was not present for the initial 60 s. *p < 0.05, **p < 0.01, ***p < 0.001. Error bars are the SEM. Data were taken from both groups 1 and 2. The number in brackets of the log is the relative length of the path taken by the animal [taken path (T)/shortest path (S)], with 2 indicating that the path was twice as long as the shortest possible path.

Different effects of previous knowledge could be observed in the resulting data, so, next we will focus on specific sessions and trials to highlight some of these effects. The simplest effect is already seen in the first GL during the Build-Up, where a significant session effect indicates that each session benefits from the experience of the previous session (groups 1 and 2: *n* = 16, *F*_(6,90)_ = 5.6, *p* < 0.001; Fig. 5*A*). This simple learning effect, while often not considered as a previous knowledge effect, does affect session performance and, thus, must be considered even in experiments that just focus on each session individually, as seen in most electrophysiological experiments (Roux et al., 2017; Lopes-Dos-Santos et al., 2018; Michon et al., 2019).

The second previous knowledge effect can be evaluated by how well an animal can navigate within an environment and how fast this navigational capability can be adapted to a new goal as soon as it has learned a specific task. Here, this was tested at every GL switch from the beginning of the Build-Up to the end of the Updates (groups 1 and 2, *n* = 16). Including the first two sessions of the first two GLs during the Build-Up as well as during the Updates (averaged across all types) revealed three distinct steps (Fig. 5*B*; session: *F*_(1,15)_ = 12.6, *p* = 0.003; GL: *F*_(2,30)_ = 8.3, *p* = 0.001; interaction: *F*_(2,30)_ = 3.9, *p* = 0.031). For the first GL, performance does not increase from the first session to the next, but, as seen in Figure 5*A*, a performance improvement develops over seven sessions. After the first GL switch (GL1 to GL2), performance decreases to the level of performance during the first session of GL1. However, a significant improvement is exposed already for the second session of GL2 (3 weeks after training start). Finally, as a third step, we find that these improvements occur in any first Update session, including additional gains in the second Update sessions (12 weeks after training start). These effects are visible across all-trial performance measurements and are likely a result of a mix of learning set effects (Harlow, 1949) as well as of an effect of increased knowledge of the maze layout. When averaging the performance across all sessions (Fig. 5*C*; groups 1 and 2, *n* = 16), animals overall had already reached plateau performance at the second GL switch during the Build-Up.

By focusing in more detail on the first and second sessions during the Updates alone, we can consider the amount of information animals need to incorporate during the Updates (groups 1 and 2, *n* = 16). We found a significant main effect of session and an interaction between session and Update types (session: *F*_(1,15)_ = 26.1, *p* < 0.001; type: *F*_(2,30)_ = 2.9, *p* = 0.072; interaction *F*_(2,30)_ = 6.3, *p* = 0.005). The follow-up test revealed that within session 1 the amount of novel information that needs to be integrated into the existing map affects the within-session online performance (just a barrier, just a new location, or both; linear contrast in S1, *p* = 0.003; Fig. 5*D*). However, this difference is eliminated by the second session, indicating that the information had been completely incorporated during the offline period.

**Figure 6.**
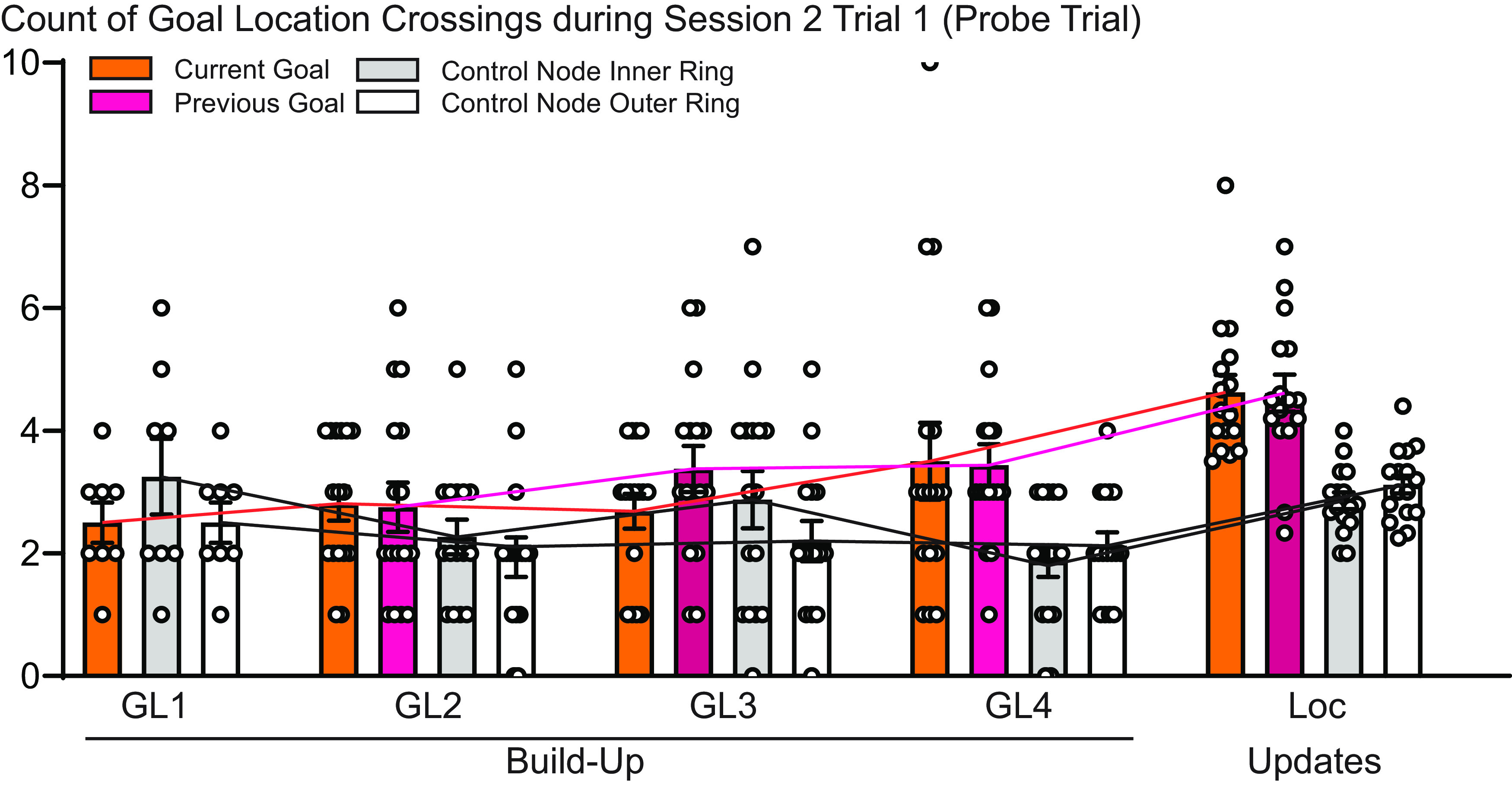
Probe trial analysis (each session 2, trial 1). Across the goal location switches during Build-Up and during the Updates, an increase in the number of crossings could be seen for both the current and previous goal locations compared with the two control nodes (groups 1 and 2, GL2–4 and Loc for full model, *n* = 16; node: *F*_(3,45)_ = 22.3, *p* < 0.001; GL2–4/Loc: *F*_(3,45)_ = 10.7, *p* < 0.001; interaction: *F*_(9,135)_ = 2.0, *p* = 0.044).

As a final step, we tested for the enhancement of long-term memories by comparing the same two sessions but only including the first trial. Similar to the all-trial performance measurement, the first session performance was worse for conditions including a GL switch (Loc and L + B) compared with just a barrier switch, but this difference disappeared by the second session (Fig. 5*E*; groups 1 and 2, *n* = 16; session: *F*_(1,15)_ = 14.4, *p* = 0.002; type: *F*_(2,30)_ = 7.2, *p* = 0.003; interaction: *F*_(2,30)_ = 1.2, *p* = 0.3). Finally, to investigate whether this enhancement of long-term memory after one-session learning was missing initially during Build-Up, the first trial performance during the second session of the Build-Up was compared with the first trial during the second session of the Updates (only Loc and L + B). This revealed a significantly better long-term memory in the second session in the Updates compared with the Build-Up (Fig. 5*F*; groups 1 and 2: *n* = 16, *t*_(15)_ = 2.1, *p* = 0.049). To confirm this effect with a different performance parameter, we counted the number of crossings for the new goal location, the previous goal location, and two control nodes (one in the inner ring of the maze, one in the outer ring) during this trial, since, because it was a probe trial, no food was present during the first trial of each second session. As can be seen in Figure 6, animals crossed both the current and last goal locations significantly more often than the control nodes starting with the second goal location, and an additional increased number of crossings were seen during the Update phase (groups 1 and 2 GL2-4 and Loc Update for full model, *n* = 16; node: *F*_(3,45)_ = 22.3, *p* < 0.001; GL2-4/Loc: *F*_(3,45)_ = 10.7, *p* < 0.001; interaction: *F*_(9,135)_ = 2.0, *p* = 0.044). Interestingly, this analysis also highlighted that animals did retain the memory of the old goal location after a goal location switch, since they tended to go more often to both the current, new goal location as well as the last goal location compared with control nodes.

### How updates affect path length

**Figure 7.**
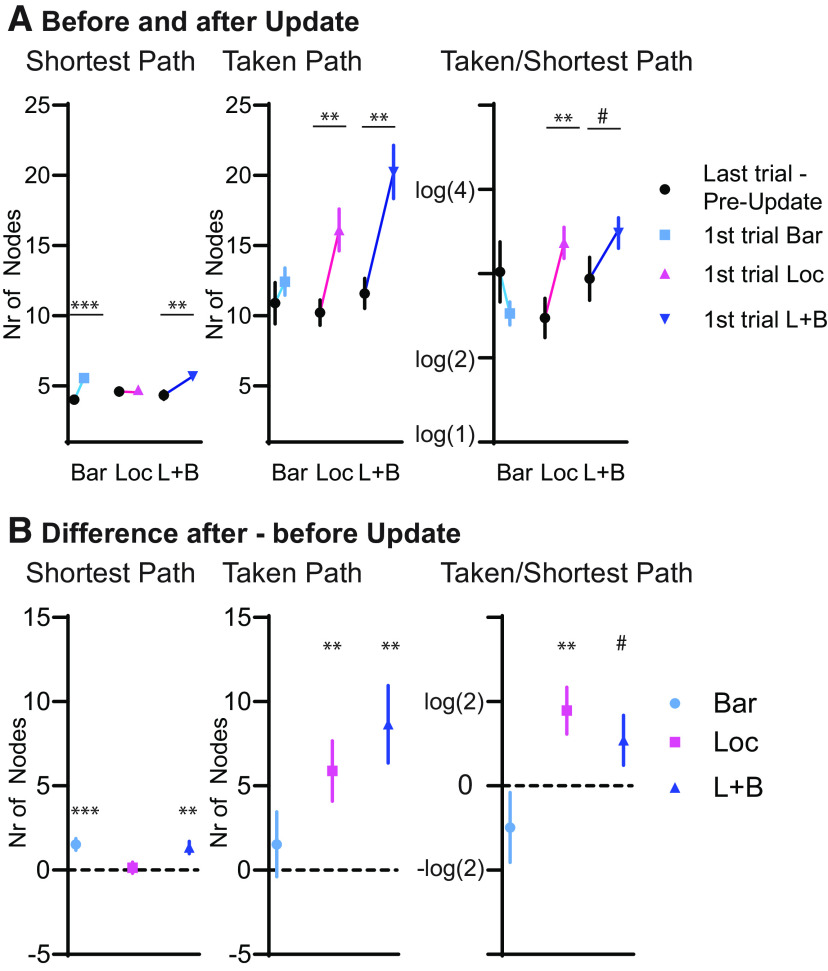
Changes because of updates. ***A***, ***B***, Always the last trial before an update (S3 of previous condition) and the first trial of the update (***A***) and the difference values (subtraction) for these (***B***). From left to right the shortest possible path, the taken path, and the relative path are presented. If barriers were included (Bar and L + B), the shortest possible path would increase from the previous trial. But only if location was changed (Loc and L + B) did the taken path increase, for the Bar update the taken path only increased by the same amount of the shortest path (two nodes). Interestingly, because of the change in shortest path, the relative change (taken/shortest) actually decreased in the Bar update. #*p* = 0.087, **p < 0.01, ***p < 0.001; ***A***, paired *t* tests; ***B***, one-sample *t* test to 0. Data taken from both groups 1 and 2. Error bars are the SEM. The number in brackets of the log is the relative length of the path taken by the animal [taken path (T)/shortest path (S)], with 2 indicating that the path was twice as long as the shortest possible path.

To characterize how the Updates themselves affect path length, the path lengths (in terms of the number of nodes) for the shortest path and the taken path are shown in Figure 7, and the normalized path length (log of taken/shortest path) is shown as used in the other figures for both the final trial before an update (usually, a session 3) and the first trial of the Update. If a barrier was included (Bar and L + B Update), there was a significant change in the shortest possible path (Bar: *t*_(15)_ = 4.39, *p* = 0.001; L + B: *t*_(15)_ = 3.69, *p* = 0.002), indicating that the inclusion of barriers does change the overall map geometry in the maze. However, the taken path only showed a significant change if the goal location was changed (taken path: Loc, *t*_(15)_ = 3.29, *p* = 0.005; L + B, *t*_(15)_ = 3.77, *p* = 0.002; normalized path: Loc, *t*_(15)_ = 3.20, *p* = 0.006; L + B, *t*_(15)_ = 1.83, *p* = 0.087). This emphasizes again that only the inclusion of barriers did not affect the performance of the mice and that the animals could rapidly adapt to this change, as was also seen in Figure 5.

### Within-session learning

**Figure 8.**
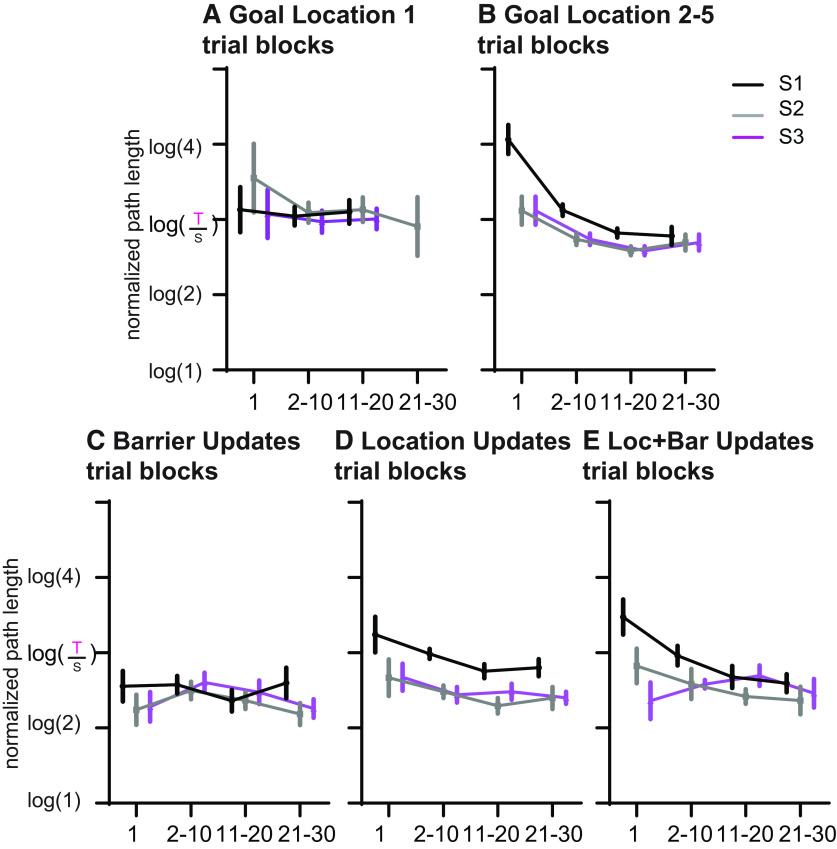
Within-session learning. ***A–E***, The change in performance within session (trial 1 and then blocks of 10 trials) always across the first three sessions (S1–3), the very first goal location (***A***), averaged across the subsequent goal locations of the buildup (***B***), for barrier updates (***C***), for location updates (***D***), and for location and barrier (Loc + Bar) updates (***E***). The first goal location did not show strong within-session learning during these first sessions; in contrast, later on (***B***) the main learning occurred between trial 1 and the next trial block during session 1 and trial 1 of sessions 2 and 3 started lower, but additional within-session improvement could be seen in the next block. In the barrier updates, performance was starting trial 1 of first session well and remained stable. For the other updates, a linear improvement during session 1 was seen across trials, and now performance was sustained to sessions 2 and 3 with no strong additional gains from the first trial to subsequent trials. For statistics, see the main text. Data were taken from both groups 1 and 2. Error bars are the SEM. The number in brackets of the log is the relative length of the path taken by the animal [taken path (T)/shortest path (S)], with 2 indicating that the path was twice as long as the shortest possible path.

To measure within-session learning, and to enable comparison across different phases of learning, trials were binned into trial blocks with trial 1, trials 2–10, trials 11–20, and trials 21–30. This was done for S1–3 of the first goal location and goal locations 2–5 in the Build-Up as well as in each Update type (Fig. 8). Since for the first goal location very few animals managed >20 trials in each session, for the overall analysis we included only up to 20 trials. There was a significant effect of training phase, session, trial block, as well as interactions between training phase and session, training phase and trial block, as well as between session and trial block (phase: *F*_(4,60)_ = 12.6, *p* < 0.001; session: *F*_(2,30)_ = 21.2, *p* < 0.001; trial block: *F*_(1.3,18.8)_ = 8.9, *p* = 0.001; phase × session: *F*_(8,120)_ = 3.7, *p* = 0.001; phase × trial block: *F*_(4.2,63.1)_ = 2.6, *p* = 0.041; session × trial block: *F*_(2.5,37.5)_ = 5.0, *p* = 0.008). For the first goal location, neither session nor trial block showed a significant effect (*p* > 0.39; Fig. 8*A*) in contrast to the subsequent goal locations of the Build-Up, during which each factor as well as the interaction showed a significant effect (session: *F*_(2,30)_ = 30.8, *p* < 0.001; trial block: *F*_(1.6,23.4)_ = 13.2, *p* < 0.001; session × trial block: *F*_(3.4,50.5)_ = 7.8, *p* < 0.001; Fig. 8*B*). This emphasizes that while the first goal location did not show strong within-session learning during these first three sessions, for the subsequent goal locations during Build-Up the main learning occurred between trial 1 and the next trial block during session 1 and trial 1 of sessions 2 and 3 started lower but additional within-session improvement could be observed in the next block. During the Updates of Loc and L + B, a linear improvement during session 1 was seen across trials, and now performance was sustained to session 2 and 3 with no strong additional gains from the first trial to subsequent trials. Thus, Loc showed significant effects of session and trial block but no interaction (session: *F*_(2,30)_ = 15.3, *p* < 0.001; trial block: *F*_(1.9,29.3)_ = 6.5, *p* = 0.004; session × trial block, *p* > 0.79; Fig. 8*D*), and for location and barrier Updates the interaction became significant as well (session: *F*_(2,30)_ = 6.4, *p* = 0.005; trial block: *F*_(3,454)_ = 4.0, *p* = 0.013; session × trial block: *F*_(6,90)_ = 3.6, *p* = 0.003; Fig. 8*E*). In contrast, the performance of barrier Updates started trial 1 of the first session well and remained stable, resulting in no significant effect of any factor or interaction (session, *p* = 0.07; other, *p* > 0.2; Fig. 8*C*).

### Other performance parameters

**Figure 9.**
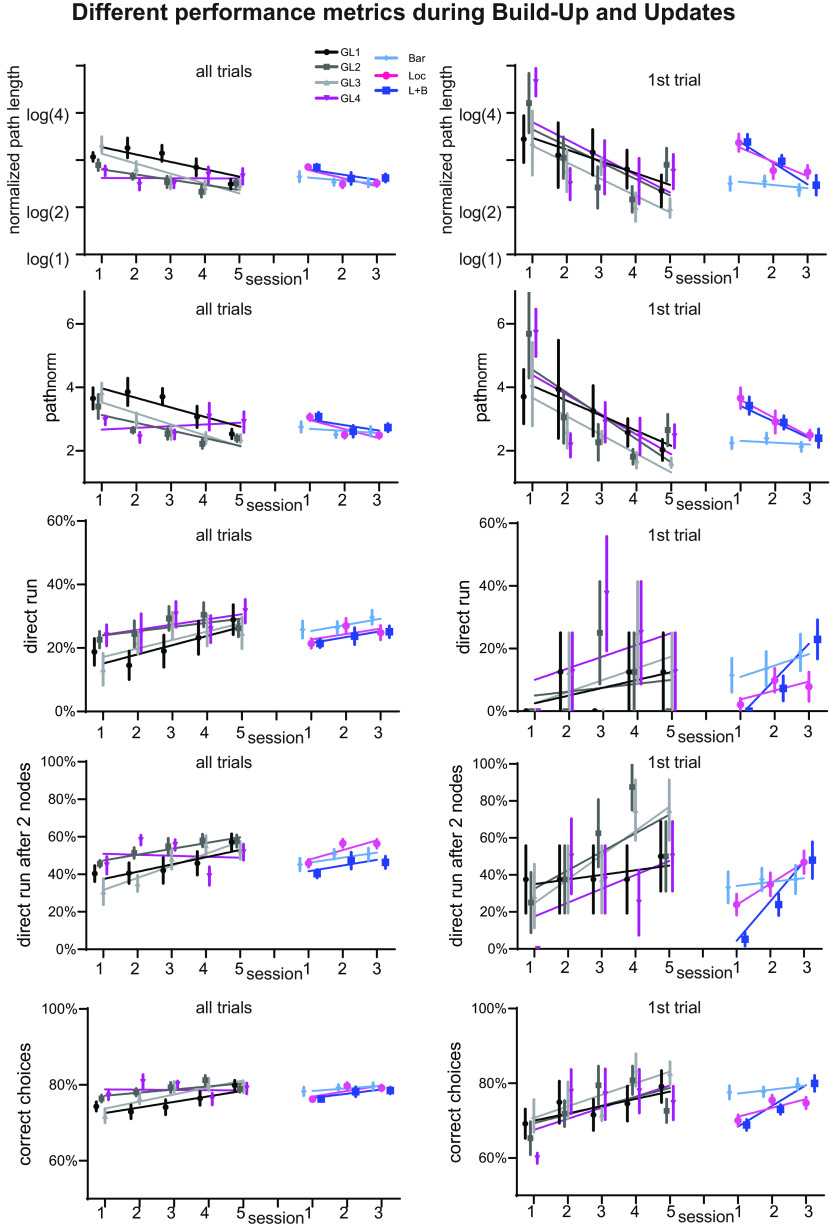
Different performance parameter buildup and updates. Shown are the log of normalized path length (top row, as used throughout the article), normalized path length, percentage of trials that was a direct run (second from top), percentage of trials that was a direct run after the second node since mice often would initially run in heading direction and then stop to consider where to go (third from top). As a final parameter, we took for each node whether the choice would bring the mouse closer to the food (correct) or not (incorrect) and created an average per trial across all traversed nodes. Each left, all trials; right, first trials for Build-Up (Group 2) and Updates (all). Error bars are the SEM. Lines are polynomial fits. The same effects seen in the log of the normalized path length can also be seen in the other parameters.

In the present analysis, we focused on using the normalized path length (the taken path in number of nodes divided by the shortest path), and since these values showed a strong skew, we used the log thereof to enable using GLM. However, other parameters, such as the normalized path length without the log transformation, the percentage of trials that were a direct run, the percentage of trials that were a direct run after the second node (since mice often would initially run in a heading direction and then stop to consider where to go), and the percentage of correct choices (Fig. 9), could also be extracted from our dataset. For the percentage of correct choices, we analyzed for each node whether the choice would bring the mouse closer to the food (correct) or not (incorrect) and created an average per trial across all traversed nodes. As can be seen in Figure 9, the same effects seen in the log of the normalized path length can also be seen in the other parameters. And while only ∼30% of the trials showed a direct run from the starting location, ∼60% of the trials showed a direct run two nodes after the starting location. In the accompanied data table, we share the raw data of all trials with all these different variables that can be used by others for further, more detailed investigations into spatial learning in mice.

## Discussion

In the present study, we aimed at developing a new rodent task that enables the investigation of map learning on memory encoding and consolidation. More specifically, we tested how mice build up and update knowledge of a large spatial map and how their navigation abilities change over time. We could show that mice learn this complex spatial map in the following three main phases: (1) learning the initial goal location, (2) faster learning after 2 weeks when learning a new goal location, and then finally (3) a third phase after 12 weeks during which they express one-session learning leading to long-term memory. The data from the HexMaze allow the investigation of many different aspects of spatial navigation and memory. Here, we focused on previous knowledge effects on performance and learning. These effects ranged from simple day-to-day performance increases to effects reflected by offline consolidation and online learning. Initial application reveals that this task can be used to test different aspects of memory while simultaneously controlling for difficulty of learning across each phase in training: from the buildup of knowledge to updates testing both across-session as well as between-session performance development. The data from the >30,000 trials are supplied with this article and can be used for many more investigations and analysis of spatial navigation in mice. In the accompanying article (Vallianatou et al., 2020), the data were used to model the strategies used by mice in the task.

How do we build up and use knowledge of a large spatial environment or map? And how will experience in a maze shape new learning? Previous knowledge will affect behavior and learning (Bartlett, 1932; Harlow, 1949; Tse et al., 2007), and, thus, needs to be considered when applying any particular training paradigm. To test how mice learn a map, we used a large spatial environment, more naturalistic in its complexity. Mice were trained to find a food location from different starting points in a maze, thereby enforcing allocentric learning to one fixed goal location per session over two training phases: Build-Up (12 weeks) and Updates (9 weeks). During the Build-Up, the goal location was kept constant for five to seven sessions before switching to a new one, while during the Updates switches occurred every three sessions. The difference between the Build-Up and Update phases is characterized by how quickly new information could be incorporated into the spatial map and thus influence the navigational behavior of the mice. Three different types of updates were introduced during this final phase: including barriers blocking certain paths, changing the goal location, and including both new barrier locations and new goal locations. Across all phases, memory effects were revealed, reflected by performance increases from one session to the next (measured in the normalized path lengths). Further, four previous knowledge effects modulating performance and learning of the spatial map are highlighted. Thus, we could show how different spatial map knowledge properties are developed stepwise over learning and could identify three main phases of learning.

### Highlighting some previous knowledge effects

The simplest and most obvious previous knowledge (or memory) effect of the spatial map is already visible in the first few sessions of the Build-Up where navigation to the invariable goal location becomes more efficient from one day to the next. This simple memory effect is what most rodent memory tasks would capture [e.g., using a radial-arm maze (Jarrard, 1995) or a watermaze, testing reference memory (Morris et al., 1982)]. While one could argue whether this simple spatial memory effect is a “previous knowledge” effect, it is important to consider it as its simplest form: knowledge gained in previous training days affects performance the succeeding day.

The second previous knowledge effect of learning a spatial map is found when comparing the performance for the very first goal location with the performance after the first and other goal location switches. Already the second goal location exposed a significant improvement in overall navigational performance to the goal location within the known map during the second session compared with the first, thus resulting in a different learning curve across sessions when comparing with the performance for the very first goal location. This change characterizes the second phase of general map learning. This effect is then enhanced once again during the Updates as performance improvement is already present in the first session and is maintained from the first to second session as well. However, this initial effect during the second goal location did not yet translate to good performance on the first trial of the second session; thus, no one-session updating leading to long-term memory was seen this early in learning. This is reminiscent of the learning set effect (Harlow, 1949). The results obtained in the HexMaze indicate that this learning-set effect can be expressed in three phases: (1) naive, (2) gains after offline consolidation, and (3) online as well as offline gains in the final stage. However, it remains unclear whether this is the result of the animals learning the rule (there is one constant food location) or the general spatial map, but most likely it is a mixture of both.

The third previous knowledge effect on spatial map updating is tied to the third phase of the learning set effect (corresponding to online gains) and is present across the different Update types: the amount of new information incorporated into the map affected how rapid online learning could occur during the first session of each update. When only the general maze structure was changed (inclusion of barriers), the animals were able to rapidly adapt their routes to the goal and additional sessions were not needed to reach optimal performance. In contrast, when the goal location or both goal location and the maze structure (L + B) were manipulated, online learning was slower, resulting in a performance decrease during the first session (linear relationship with the number of elements changed). However, offline consolidation eliminated this effect and by the second session animals performed similarly for all Update types. This effect could potentially be linked to a schema or schema-like effect of the knowledge of the cognitive map. Considering the degree of change compared with the previously learned information could explain some differences in schema effects in previous rodent and human studies. In the original paired-associate task (Tse et al., 2007), the hippocampus was necessary during update encoding, and this hippocampal involvement was also observed in a similar human schema task testing for a recently acquired, simple schema (card–location associations; van Buuren et al., 2014). In contrast, during human schema tasks that involve long-established, real-world schemas, the hippocampus tends not to be active, and instead the prefrontal cortex directly communicates with the other cortical regions (van Kesteren et al., 2010a,b, 2012). It would be tempting to speculate that there may be a gradient across the complexity or extent of an existing schema, which in combination with the amount of new information overlap, results in a shift from hippocampal to cortical involvement (Alonso et al., 2020). (1) If no schema is present, the hippocampus is necessary for weeks to months; (2) if a simple schema is present, the hippocampus is necessary for memory encoding but new information becomes more rapidly hippocampal independent; and (3) if a complex schema is present, the hippocampus is not even necessary for encoding, similar to fast mapping (Coutanche and Thompson-Schill, 2014, 2015; but also see Cooper et al., 2019). For a more detailed review of this concept, please see the study by Alonso et al. (2020).

The fourth previous knowledge effect of knowing the spatial map is reflected in long-term memory performance (first trial of each session) and is the critical difference between our Build-Up and Update phases, and thus is indicative of the third phase of spatial map learning. Initially, during the Build-Up, the animals show poor long-term memory (2–3 d) after one training session to a goal location; during the Updates, the consistent development of long-term memory is accelerated and detectable in the probe trials (critical trial for this is the first trial of the second session). Interestingly, counting the crossings of both the new as well as the last goal location revealed that animals retained the memory of the last goal location as well as learning the new one. Thus, new information did not overwrite the old information. However, one training session only led to a 2 d and not 5 d memory here in mice. For long-term memory to last 5 d in mice, two training sessions were required. This acceleration of consolidation has previously been linked to the schema effect (Tse et al., 2007), and therefore it could be speculated that the knowledge of the map may be linked to schema or schema-like effects.

The HexMaze also revealed interesting features of map effects in mice. First, we are the first to show that the Build-Up of the cognitive map is dependent on time but not training or experience. This was revealed by training animals either two or three times a week. When comparing these two training conditions, performance was more similar when aligned to time (weeks since start of training) than to the number of days already spent in training. Further, after the 12 week Build-Up with either 36 or 24 sessions of training, all animals showed rapid consolidation during the Updates, confirming the established cognitive map was independent of training amount. Thus, time dependency, and not experience dependency, indicates that the buildup of a knowledge network requires a remodeling of the network, which, importantly, occurs offline and for a certain time period and cannot be facilitated by a training increase. This is reminiscent of the massed versus spaced memory effect: massed training creates a stronger initial memory; however, spaced training creates a memory trace that lasts longer (da Silva et al., 2014; Nonaka et al., 2017).

### Schema versus learning set

Can a cognitive map, as tested in the HexMaze, be considered as a schema? There are many definitions of schema, as we recently reviewed (Alonso et al., 2020). Human schema investigations have used different types of schema from spatial maps of object–location pairs (van Buuren et al., 2014); semantic concepts (van der Linden et al., 2017); visual–texture combinations (van Kesteren et al., 2010b), and movies (van Kesteren et al., 2010a). In contrast, many rodent studies have used the term schema more loosely [e.g., to describe the first experience with a linear track (Dragoi and Tonegawa, 2013) or a daily changing sequence of goal locations on a circular track (McKenzie et al., 2013)]. Recently Ghosh and Gilboa (2014) summarized the following four key features of schemas: (1) an associative network structure, (2) based on multiple episodes, (3) a lack of unit detail, and (4) adaptability. The requirements are present in our task for testing a spatial map: the multiple extramaze and intramaze cues together with the maze layout represent the associate network structure; training takes multiple sessions or episodes; and we have shown adaptability in the Updates. However, we did not test the same animals in a similar maze with different extramaze cues. Further, animals could have used episodic memory of the last event/trial to solve the task, although by using different starting points in each trial we ensured that each trial did have a different path. At this point, it remains disputable whether the task does test extracted commonalities and shows a lack of unit detail. It is possible that the animals used specific features of the maze, rather than an abstract and general knowledge and therefore schema. Therefore, while it is tempting to speculate that in this task the map of the environment acts as a schema, currently there is not enough evidence for this. What we could show is that knowledge of the map after 12 weeks of learning led to expedited long-term memory. Expedited long-term memory has been argued to be a key feature of schemas (Ghosh and Gilboa, 2014; Fernández and Morris, 2018; Alonso et al., 2020).

Another argument that spatial maps in general can be seen as schema is that they use the same underlying mechanisms. With place cells in the hippocampus and grid cells in the entorhinal cortex, we have learned about the basic building blocks for how the cognitive map is coded in the brain (McNaughton et al., 2006; Moser et al., 2008). These same fundamental building blocks have been shown to then also be harnessed for nonspatial memory representation and associations between these (Behrens et al., 2018). Therefore, in general, map learning can be the ideal model for us to understand how we build up as well as update our knowledge systems and therefore schemas.

One criticism of schema tasks such as the paired-associated task is that usually pretraining on the schema and the updates differ in difficulty and cognitive load because the amount of items learned was differed in the build-up versus the update (Tse et al., 2007; van Buuren et al., 2014), which could account for the rapid updating effect that is the hallmark of schemas. The advantage of our framework is that during both the Build-Up and Updates only one goal location is presented for multiple sessions, thereby keeping the task difficulty constant.

Another previous knowledge effect described in the literature is learning sets (Harlow, 1949). The difference between learning sets and schemas is that learning sets describe learning a set of rules that can be applied to new information. This is in contrast with schemas that are an associated network structure that can accommodate new learning. Our task-learning set would be the animal learning about the principle that there is one goal location within the maze that stays constant for a certain amount of time but then can change. We believe that this effect can be seen when the animal is learning the first and second goal locations during buildup.

### How the task can be applied

The three different phases in the HexMaze are optimal to apply to different types of experiments. For example, if the goal is to test classic reference memory, simply using the first seven sessions to the goal location is sufficient. In contrast, if the aim would be to measure neural correlates of navigation within an environment with many days of data for direct comparisons, training should first be to one goal location, but analysis would be applied from the second session of the second goal location onward when performance is stable over time (i.e., from the ninth training day). As a third application example, the investigation of offline memory consolidation would occur during the Updates as here, each change is comparable to the next (plateau performance). One key advantage of the HexMaze to many other rodent tasks is the following: because of the naturalistic paradigm, mice rapidly habituate to the maze (two 1 h sessions of habituation with all cage mates at once primarily for stress-free pickups with tubing) and do not require other pretraining/shaping.

One noticeable challenge in the behavior of the mice in the maze, is that they never reached perfect performance. Instead, even when a specific goal location was experienced for multiple sessions, the mice only performed perfectly with direct runs from start in ∼30% of the trials, which increased to ∼60% if you considered performance after the animals passed the first two nodes. This lack of perfect goal-oriented behavior from the starting location may be because of the difficulty of the task, but more likely is because of the nature of the species itself. In contrast to rats, mice move rapidly in bursts and show more shuttling and random movements, which is likely inert behavior to avoid predators (Jones et al., 2017), and even in known environments use random movement strategies (Gire et al., 2016). The prevalence of random movement patterns could be confirmed in the HexMaze by using a modeling approach to the data (Vallianatou et al., 2020). Instead of increased goal-directed behavior from the starting location, learning is expressed in increased foresight: the point of direct run to the goal location will move further away from the goal as experience with the maze increases. However, importantly the modeling approach also confirmed that the behavior of the mice in the HexMaze is better than a random run through the maze once they learned the goal location (Vallianatou et al., 2020). We are currently developing a rat version of the HexMaze and can confirm that rats show much more goal-oriented behavior in the maze than mice.

### Conclusion

In sum, we have developed a flexible rodent task in which different effects of previous knowledge of a spatial map on navigational and memory performance, encoding, and updating can be investigated and both offline long-term memory and online navigational performance can be evaluated separately. We could show that mice learn this complex spatial map in the following three main phases: (1) learning the initial goal location, (2) faster learning after 2 weeks when learning a new goal location, and then finally (3) a third phase after 12 weeks to express one-session learning leading to long-term memory. We have highlighted different effects that can be seen in this very rich dataset with >30,000 trials, here focusing on the metric of normalized path length and previous knowledge effects. However, many more metrics such as binary choices at each node and the presence of direct runs are provided in the dataset as well. Thus, the dataset (Extended Data Fig. 1-1) can be used for many other applications and investigations into mouse navigation, as also seen in the accompanying article (Vallianatou et al., 2020).

Further, the task itself will enable future studies investigating the principles of memory updates and the involved mechanisms. While we have not yet investigated whether the effect of rapid systems consolidation (hippocampal independency) is present in this task as well, we did find a behavioral rapid updating effect that is likely to be accompanied by the consolidation effect. Overall, our brains are tuned to remembering things that are new, but how novel something is will depend on our experiences (Duszkiewicz et al., 2019).

## Synthesis

Reviewing Editor: Carmen Sandi, Swiss Federal Institute of Technology Decisions are customarily a result of the Reviewing Editor and the peer reviewers coming together and discussing their recommendations until a consensus is reached. When revisions are invited, a fact-based synthesis statement explaining their decision and outlining what is needed to prepare a revision will be listed below. The following reviewer(s) agreed to reveal their identity: NONE.

One of the original reviewers and the review editor have assessed the revised manuscript and consider it to be improved. There are a few remaining issues that still need attention:

The reviewer acknowleges that the authors do not agree with their definition of a schema, which is “a generalizable cognitive construct that can be used by subjects to solve a related but new problem", yet have no problem with the four key features of a schema proposed by Gosh and Gilboa. But their definition is just a reiteration of Gosh and Gilboa's words.

For one of the four key features - “basis on multiple episodes” - Gosh and Gilboa wrote: “Schemas are general, higher-level constructs that encompass representations of the similarities or commonalities across events, rather than the specificity that make those events unique.” Besides, in the same review paper written by Gosh and Gilboa, “generalize” or “extracted commonalities” are used in many places to describe a schema. For example:

"That schemas are composed of extracted commonalities across events relies on two necessary features: (1) basis on multiple episodes and (2) lack of unit detail.”

"When participants were presented with partial patterns, ... they were able to generalize their prior knowledge to the new situation to inform their decision.... By our interpretation, ... the partial patterns cued activation of the appropriate schema.”

"Ideas about schemas were also refined in the perceptual literature where the extraction of commonalities among a set of patterns played a central role.”

"Episodic memory is memory for a specific autobiographical event, or episode, and so is quite distinct from schemas that are constantly developing over experiences through extraction of commonalities.”

Based on above statements, the reviewer disagrees with the authors that “the knowledge of the map of the HexMaze is the schema” because the knowledge about the map could encompass specific information including the intra- and extra-maze cues, which is more like an “episodic” memory rather than an “abstract” or “semantic” schema. Although the reviewer contemplates the possibility that mice could form a schema memory about the maze, they eed to see concrete evidence, especially for distinction from other types of memory.

Such distinction is critical but not clear from this study because there is only one maze in the task. The mice’s performance can totally depend on how well they know the *specific* features of the maze, rather than an abstract and general knowledge or a schema of a “HexMaze”. Again, the reviewer tends to believe the mice learn some sort of a schema of the maze even there is only one, but to test for the existence of a schema, another HexMaze that has a similar layout or at least the same layout but with different sensory cues would be needed. A good example of this can be seen in recent work by Baraduc et al., Science 2019 where multiple similar but different mazes are used to find single-unit correlates of a spatial schema in the hippocampus. Baraduc et al would be able to claim they have found “schema cells” because some hippocampal neurons show highly correlated firing in different but similar mazes, but not because these neurons have specific place fields in one particular maze.

The authors claim that the schema of the HexMaze is based on multiple episodes (one key feature of schemas) because training takes multiple sessions. If each repeated session can be seen as a different episode, almost all behavioral training tasks can be seen as a “schema” task since training almost always takes multiple sessions. So there are must be significant differences between these “episodes” so the use of schema does not become trivial. For this particular task, only changing food locations across sessions does not seem to be significant differences to me since the specific maze is still the same.

The authors talked about the analogy between the current task and the paired-associates (PA) task. The reviwer thinks there are drastic differences between the two. The schema in the PA task is not the spatial layout of the maze, but the layout of the PAs. To show there is indeed a schema effect, the Morris group uses a different but similar layout of the PAs and shows facilitated behavioral learning of new paired associates (i.e., rapid assimilation into the current schema). This kind of evidence is not seen in the current study since these mice are always trained in the same maze.

To further explain the reviewer’s understanding of what a schema is, they will use a simple example. Suppose two kids are playing a “hide-and-seek” game in a house. They are getting better at both hiding and seeking overtime but why? Apparently because over time they know better about the environment of this particular house. But will a schema of a “house” be formed in the kids’ brains? Maybe and I tend to think they do. But the reviewer is not convinced until the evidence is shown, such as that they immediately perform well in another different house, otherwise their improved performance could be explained by an increased “episodic” memory about the single house but not an abstract, general concept of houses.

There is indeed ambiguity in the usage of the term schema in literature, especially in different studies across species. The reviewer is worried that the current work would not help much clarify this important concept but instead further muddy the waters.

## Author Response

Response to Reviewers

One of the original reviewers and the review editor have assessed the revised manuscript and consider it to be improved. There are a few remaining issues that still need attention:

The reviewer acknowleges that the authors do not agree with their definition of a schema, which is “a generalizable cognitive construct that can be used by subjects to solve a related but new problem", yet have no problem with the four key features of a schema proposed by Gosh and Gilboa. But their definition is just a reiteration of Gosh and Gilboa’s words.

For one of the four key features - “basis on multiple episodes” - Gosh and Gilboa wrote: “Schemas are general, higher-level constructs that encompass representations of the similarities or commonalities across events, rather than the specificity that make those events unique.” Besides, in the same review paper written by Gosh and Gilboa, “generalize” or “extracted commonalities” are used in many places to describe a schema. For example:

"That schemas are composed of extracted commonalities across events relies on two necessary features: (1) basis on multiple episodes and (2) lack of unit detail.”

"When participants were presented with partial patterns, ... they were able to generalize their prior knowledge to the new situation to inform their decision.... By our interpretation, ... the partial patterns cued activation of the appropriate schema.”

"Ideas about schemas were also refined in the perceptual literature where the extraction of commonalities among a set of patterns played a central role.”

"Episodic memory is memory for a specific autobiographical event, or episode, and so is quite distinct from schemas that are constantly developing over experiences through extraction of commonalities.”

Based on above statements, the reviewer disagrees with the authors that “the knowledge of the map of the HexMaze is the schema” because the knowledge about the map could encompass specific information including the intra- and extra-maze cues, which is more like an “episodic” memory rather than an “abstract” or “semantic” schema. Although the reviewer contemplates the possibility that mice could form a schema memory about the maze, they eed to see concrete evidence, especially for distinction from other types of memory.

Such distinction is critical but not clear from this study because there is only one maze in the task. The mice’s performance can totally depend on how well they know the *specific* features of the maze, rather than an abstract and general knowledge or a schema of a “HexMaze”. Again, the reviewer tends to believe the mice learn some sort of a schema of the maze even there is only one, but to test for the existence of a schema, another HexMaze that has a similar layout or at least the same layout but with different sensory cues would be needed. A good example of this can be seen in recent work by Baraduc et al., Science 2019 where multiple similar but different mazes are used to find single-unit correlates of a spatial schema in the hippocampus. Baraduc et al would be able to claim they have found “schema cells” because some hippocampal neurons show highly correlated firing in different but similar mazes, but not because these neurons have specific place fields in one particular maze.

The authors claim that the schema of the HexMaze is based on multiple episodes (one key feature of schemas) because training takes multiple sessions. If each repeated session can be seen as a different episode, almost all behavioral training tasks can be seen as a “schema” task since training almost always takes multiple sessions. So there are must be significant differences between these “episodes” so the use of schema does not become trivial. For this particular task, only changing food locations across sessions does not seem to be significant differences to me since the specific maze is still the same.

The authors talked about the analogy between the current task and the paired-associates (PA) task. The reviwer thinks there are drastic differences between the two. The schema in the PA task is not the spatial layout of the maze, but the layout of the PAs. To show there is indeed a schema effect, the Morris group uses a different but similar layout of the PAs and shows facilitated behavioral learning of new paired associates (i.e., rapid assimilation into the current schema). This kind of evidence is not seen in the current study since these mice are always trained in the same maze.

To further explain the reviewer’s understanding of what a schema is, they will use a simple example. Suppose two kids are playing a “hide-and-seek” game in a house. They are getting better at both hiding and seeking overtime but why? Apparently because over time they know better about the environment of this particular house. But will a schema of a “house” be formed in the kids’ brains? Maybe and I tend to think they do. But the reviewer is not convinced until the evidence is shown, such as that they immediately perform well in another different house, otherwise their improved performance could be explained by an increased “episodic” memory about the single house but not an abstract, general concept of houses.

There is indeed ambiguity in the usage of the term schema in literature, especially in different studies across species. The reviewer is worried that the current work would not help much clarify this important concept but instead further muddy the waters.

We thank the reviewer for their detailed discussion on schemas. We now adapted the text to only refer to us testing a cognitive map and previous knowledge and not schema, instead we only discuss in one section why this could potentially be a schema. Further, we expanded that section where we discuss if this task could be seen as schema task to include the caveats mentioned by the reviewer. (see changes throughout text in tracked changes version for deletion of “schema” )

Schema versus learning set

Can a cognitive map, as tested in the HexMaze, be considered as a schema? There are many definitions of schema, as we recently reviewed (Alonso et al., 2020). Human schema investigations have used different types of schema from spatial maps of object-location pairs (van Buuren et al., 2014), semantic concepts (van der Linden et al., 2017), visual-texture combinations (van Kesteren, Rijpkema, Ruiter, & Fernandez, 2010) and movies (van Kesteren, Fernandez, et al., 2010a). In contrast, many rodent studies have used the term schema more loosely e.g. to describe the first experience with a linear track (Dragoi & Tonegawa, 2013) or a daily-changing sequence of goal locations on a circular track (McKenzie et al., 2013). Recently Ghosh and Gilboa (2014) summarized four key features of schemas: (1) an associative network structure, (2) basis on multiple episodes, (3) lack of unit detail, and (4) adaptability. The requirements are present in our task for testing spatial map: the multiple extra- and intra-maze cues together with the maze-layout represent the associate network structure, training takes multiple sessions or episodes and we have shown adaptability in the Updates. However, we did not test the same animals in a similar maze with different extra-maze cues. Further, animals could have used episodic memory of the last event/trial to solve the task, even though by using different starting points each trial we ensured that each trial did have a different path. At this point, it remains disputable if the task does test extracted commonalities and shows a lack of unit detail. It is possible that the animals used specific features of the maze, rather than an abstract and general knowledge and therefore schema. Therefore, while it is tempting to speculate that in this task the map of the environment acts as an schema, currently there is not enough evidence for this. What we could show is that knowledge of the map after twelve weeks of learning, led to expedited long-term memory. Expedited long-term memory has been argued to be a key feature of schemas (Alonso et al., 2020; Fernández & Morris, 2018; Ghosh & Gilboa, 2014)

Another argument that spatial maps in general can be seen as schema, is that they use the same underlying mechanisms. With place-cells in the hippocampus and grid-cells in the entorhinal cortex, we have learned about the basic building blocks how the cognitive map is coded in the brain (McNaughton et al., 2006; Moser et al., 2008). These same fundamental building blocks have been shown to then also be harnessed for non-spatial memory representation and associations between these (Behrens et al., 2018). Therefore, in general map learning can be the ideal model for us to understand how we build-up as well as update our knowledge systems and therefore schemas.
